# Modulation recognition method of mixed signals based on cyclic spectrum projection

**DOI:** 10.1038/s41598-023-48467-w

**Published:** 2023-12-05

**Authors:** Weichao Yang, Ke Ren, Yu Du, Jia Zheng, Yifan Ping, Sujun Wang, Xinquan Yang, Li Li

**Affiliations:** National Key Laboratory of Science and Technology on Space Microwave, China Academy of Space Technology (Xi’an), Xi’an, 710100 China

**Keywords:** Electrical and electronic engineering, Aerospace engineering

## Abstract

The signal in the receiver is mainly a combination of different modulation types due to the complex electromagnetic environment, which makes the modulation recognition of the mixed signal a hot topic in recent years. In response to the poor adaptability of existing mixed signals recognition methods, this paper proposes a new recognition method for mixed signals based on cyclic spectrum projection and deep neural network. Firstly, through theoretical derivation, we prove the feasibility of using cyclic spectrum for mixed communication signal identification. Then, we adopt grayscale projections on the two-dimensional cyclic spectrum as identifying representation. And a new nonlinear piecewise mapping and directed pseudo-clustering method are used to enhance the above-mentioned grayscale images, which reduces the impact of energy ratios and symbol rates on signal identification. Finally, we use deep neural networks to extract deep abstract modulation information to achieve effective recognition of mixed signals. Simulation results show that the proposed method is robust against noise. When signal-to-noise ratio is not less than 0 dB, the average recognition rate is greater than 95%. Furthermore, this method exhibits good robustness towards the changes in signal symbol rates and energy ratios between mixed signals.

## Introduction

Modulation recognition of communication signal is to determine the modulation mode of signal under the condition of unknown or known a small amount of signal prior information. This technology has been widely used in the fields of military electronic reconnaissance, electromagnetic spectrum warfare and civil spectrum monitoring. After years of development, modulation recognition technology has formed a relatively complete theoretical system. In recent years, with the increasing complexity of the electromagnetic environment, the receiver receives no longer a single signal, but a mixed form of two or more signals. The mixed signals have the characteristics of time–frequency domain aliasing, and many existing methods are no longer applicable^1–3^. Therefore, more and more scholars begin to pay attention to the modulation recognition of mixed signals^4–11^.

The mixture of signals will inevitably bring about the complexity of recognition feature extraction. The method based on blind source separation (BSS) is an important approach to solve mixed signal recognition. In fact, such methods still belong to the category of single signal identification, and in the absence of any prior information, the BSS method can not effectively separate the signals with complete spectral aliasing^12,13^. In addition, the BSS method is often affected by the dual effects of noise and errors introduced by front-end separation algorithms, resulting in poor recognition performance.

In recent years, the successful application of artificial intelligence methods in single signal modulation recognition has provided new ideas for this problem^14–20^. Some scholars have made many attempts and explorations in intelligent recognition of mixed signals^21–26^. Reference^21,22^ proposed a co-channel multi-signal modulation identification method based on deep learning capsule network. This type of method mainly takes advantage of the multi-mode processing and vector output of the capsule network. However, it is assumed that the signals are co-channel with different frequencies, and multiple signals are separable in the frequency domain, so they belong to the category of single-signal identification in a strict sense. References^23–25^ have all proposed co-channel mixed signal recognition methods based on convolutional neural network. The similarity is that the network input of the three methods is the time–frequency domain characteristics of mixed signals. The difference is that reference^24^ constructs multi-label convolutional neural network (MLCNN) for multi-label classification and multi-decision threshold optimization method based on output label decision, while reference^25^ makes use of the advantages of fast convergence speed and high recognition accuracy of ResNet. The advantage of this type of method is that it directly adopts the classical deep learning method to intelligently analyze and identify the time domain information of the signal. It is easily affected by noise, so the recognition robustness beyond the training coverage signal-to-noise ratio is poor. To some extent, although the reference^25^ uses the feature difference of mixed signals to construct frequency domain motion and shear image enhancement to improve the anti-noise ability of the algorithm, the improvement space is still limited. In addition, the above three methods do not consider the changes of signal parameters such as energy ratio between signals, symbol rate, etc., so these methods have poor adaptability and mobility.

Cyclic spectrum is a feature insensitive to noise, many scholars have proposed modulation recognition methods for communication signals based on cyclic spectrum^26–32^, which have good anti-noise ability. Reference^26–28^ belong to the category of single signal modulation recognition, and the influence of signal parameter transformation is not considered. The method proposed in reference^29^ needs to demodulate the signal, which is difficult to demodulate without prior information. In addition, the characteristics of the adopted signal, such as constellation quadrant distribution, frequency tracking distribution distance and baseband data distribution distance, are easily affected by noise. Reference^30^ uses cyclic spectrum projection combined with deep neural networks to identify mixed signals, and enhances cyclic spectrum projection from an image processing perspective, such as flip transformation, gray scale transformation, image clipping, resolution transformation, standardization. This method does not consider the impact of changes in image texture position caused by signal parameter changes, and has not been analyzed and explained in subsequent simulations. Reference^31^ uses joint features such as high-order cumulants and cyclic spectra to achieve modulation recognition of dual mixed signals, including signals such as 2ASK (2 amplitude shift keying), 4ASK (4 amplitude Shift Keying), BPSK (binary phase shift keying), QPSK (quadrature phase shift keying), but still does not consider the influence of parameter changes such as energy ratio and symbol rate between signals. Reference^32^ uses cyclic spectrum cross-section features to achieve modulation recognition of mixed signals, rather than cyclic spectrum projection. In fact, cyclic spectrum projection has three-dimensional feature attributes, which contain more cyclic spectrum information and can effectively characterize the differences of mixed signals. Similar to intelligent methods, the above methods based on complete cyclic spectrum information do not comprehensively consider and evaluate the influence of all interference factors, resulting in poor stability and applicability of the algorithm.

In response to the above issues, this paper proposes a deep neural network recognition method based on cyclic spectrum projection, which converts one-dimensional signal modulation recognition problems into two-dimensional cyclic spectrum image recognition problems. Firstly, we derive and analyze the cyclic spectrum characteristics of single signals and mixed signals in detail. We use grayscale projections of cyclic spectrum as data representation, and then adopt two novel feature enhancement methods, one is the nonlinear piecewise mapping which makes signal representation with different energy ratios tend towards consistency, reducing the impact of energy ratios on recognition performance; the other one is directed pseudo-clustering which eliminates spectral line spacing caused by symbol rates, avoiding effects from signal symbol rates on recognition performance. Based on enhanced cyclic spectrum grayscale images, we utilize the excellent multi-level detail extraction capabilities of residual neural network^25^ to achieve effective identification of several commonly used but difficult to distinguish phase modulated mixed signals in satellite communications. Simulation results show that the proposed method is insensitive to noise, and the average recognition rate is greater than 95% when the signal-to-noise ratio is not lower than 0 dB. Moreover, the proposed method has good adaptability to the changes in signal symbol rates and energy ratios between mixed signal.

## Cyclic spectrum characteristics of mixed signals

In this paper, the mixed modulation recognition of BPSK, QPSK, and OQPSK (offset quadrature phase shift keying) signals, is studied under the same frequency and bandwidth. These three types of modulation signals are commonly used and difficult to distinguish in satellite communications system, such as Advanced Communications Technology Satellite (ACTS), Mobile User Objective System (MUOS) and Advanced Extremely High Frequency (AEHF)^33^. The three kinds of signals have many similar characteristics in the time–frequency domain. The problem of mixed double-signal modulation recognition, especially in the case of the same frequency, the same symbol rate and the uncertainty of the energy ratio, has been one of the recognized difficulties in the field of signal recognition.

This paper attempts to solve the mixed recognition problem of the above three signals from the signal cyclic spectrum domain. This section analyzes the cyclic spectrum characteristics and differences of single signal and mixed double-signal in detail.

### Cyclic spectrum characteristics of single signal

In this paper we assume that the signal has been processed by channel correction and channel equalization. BPSK, QPSK and OQPSK signals can be expressed by a unified mathematical expression:

1 $$ x\left( t \right) = c\left( t \right)\cos \left( {2{\uppi }f_{c} t + \phi_{0} } \right) - s\left( t \right)\sin \left( {2{\uppi }f_{c} t + \phi_{0} } \right) $$

where $c\left( t \right) = \sum\limits_{n = - \infty }^{\infty } {c_{n} q\left( {t - nT - t_{0} } \right)}$, $s\left( t \right) = \sum\limits_{n = - \infty }^{\infty } {s_{n} q\left( {t - nT - t_{0} } \right)}$, $c_{n}$ and $s_{n}$ are equal probability, and the value is $\pm 1$, $q\left( \cdot \right)$ denotes rectangular pulse, $T$ denotes symbol period, and ${1 \mathord{\left/ {\vphantom {1 T}} \right. \kern-0pt} T}$ is symbol rate, $f_{c}$ represents the carrier frequency, $\phi_{0}$ denotes the initial phase.

**Figure 1:**
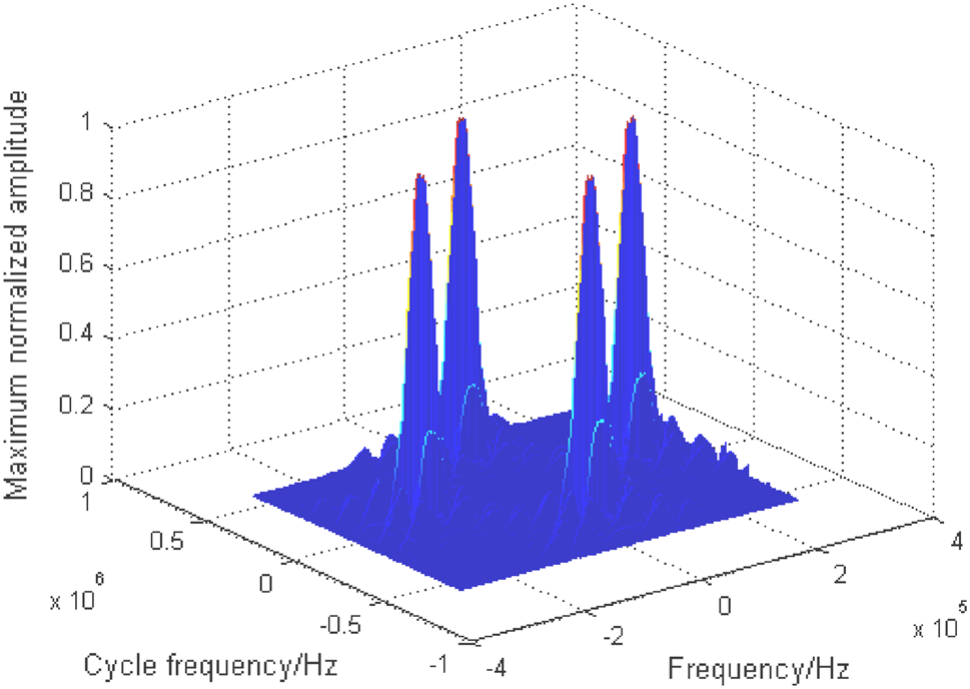
Cyclic spectrum of BPSK signal.

**Figure 2:**
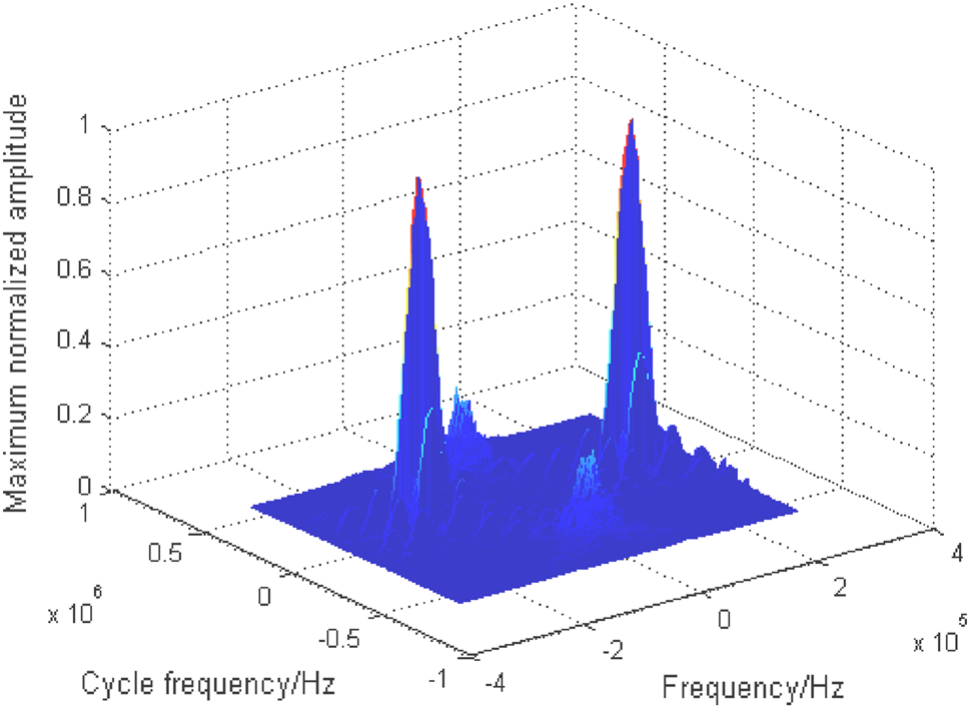
Cyclic spectrum of QPSK signal**.**

**Figure 3:**
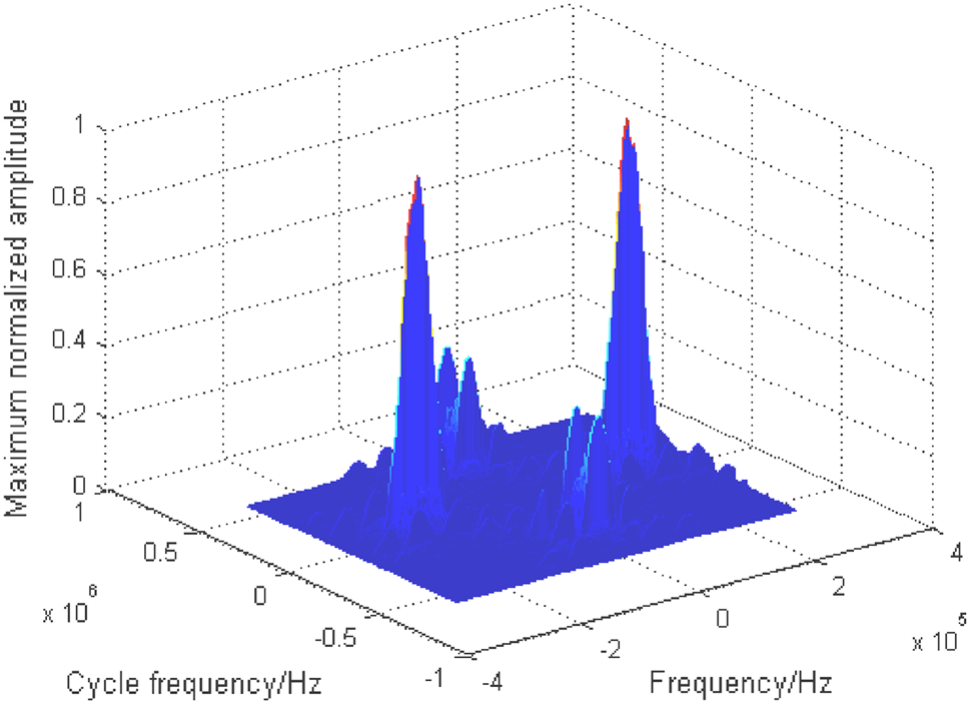
Cyclic spectrum of OQPSK signal.

For the BPSK signal, $s\left( t \right) = 0$. For the QPSK signal, $c\left( t \right)$ and $s\left( t \right)$ do not need to be changed. For OQPSK signal, $c\left( t \right) = \sum\limits_{n = - \infty }^{\infty } {c_{n} q\left( {t - nT - {T \mathord{\left/ {\vphantom {T 2}} \right. \kern-0pt} 2} - t_{0} } \right)}$. The cyclic spectrum equation of the three signals is as follows:

2 $$S_{{{\text{BPSK}}}}^{\alpha } \left( f \right) = \left\{ {\begin{array}{ll}    {\frac{E}{{4T}}\left[ {Q\left( {f - f_{c}  + \frac{\alpha }{2}} \right)Q^{ * } \left( {f - f_{c}  - \frac{\alpha }{2}} \right) + \left. {Q\left( {f + f_{c}  + \frac{\alpha }{2}} \right)Q^{ * } \left( {f + f_{c}  - \frac{\alpha }{2}} \right)} \right]} \right.\exp \left( { - {{{\rm j}2\uppi }}\alpha t_{0} } \right),\;{\text{when}}\;\alpha  = \frac{n}{T}}  \\    \begin{gathered}   \frac{E}{{4T}}\left[ {\exp \left( {{{\rm j}}2\phi _{0} } \right)Q\left( {f - f_{c}  + \frac{\alpha }{2}} \right)Q^{ * } \left( {f + f_{c}  - \frac{\alpha }{2}} \right) + \exp \left( { - {{\rm j}}2\phi _{0} } \right)Q\left( {f + f_{c}  + \frac{\alpha }{2}} \right)Q^{ * } \left( {f - f_{c}  - \frac{\alpha }{2}} \right)} \right]\exp \left( { - {{{\rm j}2\uppi }}\alpha t_{0} } \right), \hfill \\   {\text{when}}{ }\;\alpha  =  \pm 2f_{c}  + \frac{n}{T} \hfill \\  \end{gathered}   \\   \end{array} } \right. $$

3 $$ S_{{{\text{QPSK}}}}^{\alpha } \left( f \right) = \frac{E}{4T}\left[ {Q\left( {f - f_{c} + \frac{\alpha }{2}} \right)Q^{ * } \left( {f - f_{c} - \frac{\alpha }{2}} \right) + Q\left( {f + f_{c} + \frac{\alpha }{2}} \right)Q^{ * } \left( {f + f_{c} - \frac{\alpha }{2}} \right)} \right]\exp \left( { - {{{\rm j}2\uppi }}\alpha t_{0} } \right),{ }{\text{when}}{ }\;\alpha = \frac{n}{T} $$

4 $$ S_{{{\text{OQPSK}}}}^{\alpha } \left( f \right) = \left\{ \begin{gathered} \frac{E}{4T}\left[ {Q\left( {f - f_{c} + \frac{\alpha }{2}} \right)Q^{ * } \left( {f - f_{c} - \frac{\alpha }{2}} \right) + \left. {Q\left( {f + f_{c} + \frac{\alpha }{2}} \right)Q^{ * } \left( {f + f_{c} - \frac{\alpha }{2}} \right)} \right]} \right.\exp \left( { - {{{\rm j}2\uppi }}\alpha t_{0} } \right), \hfill \\ {\text{when}}{ }\alpha = \frac{n}{T},n\left( {even} \right) \hfill \\ \frac{E}{4T}\left[ {\exp \left( {{{\rm j}}2\phi_{0} } \right)Q\left( {f - f_{c} + \frac{\alpha }{2}} \right)Q^{ * } \left( {f + f_{c} - \frac{\alpha }{2}} \right) + \left. {\exp \left( { - {{\rm j}}2\phi_{0} } \right)Q\left( {f + f_{c} + \frac{\alpha }{2}} \right)Q^{ * } \left( {f - f_{c} - \frac{\alpha }{2}} \right)} \right]} \right.\exp \left( { - {{{\rm j}2\uppi }}\alpha t_{0} } \right), \hfill \\ {\text{when}}{ }\alpha = \frac{n}{T} \pm 2f_{c} ,n\left( {odd} \right) \hfill \\ \end{gathered} \right. $$

where $Q\left( f \right) = \frac{{\sin \left( {{\uppi }fT} \right)}}{{{\uppi }f}}$, *n* is an integer and $\frac{n}{T}$ is an integer multiple of the symbol rate. *E* represents the signal energy, and the three signal cycle spectrums are shown in Figs. 1, 2 and 3.

Gaussian noise is not periodic, so it mainly exists in $\alpha = 0$ section. In order to reduce the impact of noise, $\alpha = 0$ section of cyclic spectrum is shielded here and in subsequent feature analysis.

1. When $\alpha = \frac{n}{T}$, the effective values of the cyclic spectrum are mainly distributed near $f{ = } \pm f_{c}$, and the cyclic spectrum is symmetric, so only this range $\left( {\alpha { > }0{,}f \ge 0} \right)$ is analyzed, and other regions also have similar characteristics.

1. When $$\alpha = \frac{{1}}{T}$$, $$f{ = }f_{c}$$
  $\left| {S_{{{\text{BPSK}}}}^{{{{1} \mathord{\left/ {\vphantom {{1} T}} \right. \kern-0pt} T}}} \left( {f_{c} } \right)} \right|{ = }\left| {S_{{{\text{QPSK}}}}^{{{{1} \mathord{\left/ {\vphantom {{1} T}} \right. \kern-0pt} T}}} \left( {f_{c} } \right)} \right| \approx \frac{E}{4T}\left| {Q\left( \frac{1}{2T} \right)} \right|^{{2}} \approx \frac{TE}{{{\uppi }^{2} }}$, OQPSK signal does not have theoretical value at this cyclic frequency.
2. When $$\alpha = \frac{{1}}{T}$$, $$f{ = }f_{c} \pm \frac{\alpha }{{4}}$$.
  $\left| {S_{{{\text{BPSK}}}}^{{{{1} \mathord{\left/ {\vphantom {{1} T}} \right. \kern-0pt} T}}} \left( {f_{c} \pm \frac{\alpha }{{4}}} \right)} \right|{ = }\left| {S_{{{\text{QPSK}}}}^{{{{1} \mathord{\left/ {\vphantom {{1} T}} \right. \kern-0pt} T}}} \left( {f_{c} \pm \frac{\alpha }{{4}}} \right)} \right| \approx \frac{E}{4T}\left| {Q\left( {\frac{3}{{{4}T}}} \right)} \right|\left| {Q\left( {\frac{1}{{{4}T}}} \right)} \right| \approx \frac{{{2}TE}}{{3{\uppi }^{2} }}$, OQPSK signal does not have theoretical value at this cyclic frequency.
3. When $$\alpha = \frac{{1}}{T}$$, $$f{ = }f_{c} \pm \frac{\alpha }{{2}}$$.
  $\left| {S_{{{\text{BPSK}}}}^{{{{1} \mathord{\left/ {\vphantom {{1} T}} \right. \kern-0pt} T}}} \left( {f_{c} \pm \frac{\alpha }{{2}}} \right)} \right|{ = }\left| {S_{{{\text{QPSK}}}}^{{{{1} \mathord{\left/ {\vphantom {{1} T}} \right. \kern-0pt} T}}} \left( {f_{c} \pm \frac{\alpha }{{2}}} \right)} \right| \approx \frac{E}{4T}\left| {Q\left( \frac{1}{T} \right)} \right| \approx {0}$, OQPSK signal does not have theoretical value at this cyclic frequency.
4. When $$\alpha = \frac{{1}}{T}$$, $$f{ = }f_{c} \pm \frac{{{3}\alpha }}{{4}}$$.
  $\left| {S_{{{\text{BPSK}}}}^{{{{1} \mathord{\left/ {\vphantom {{1} T}} \right. \kern-0pt} T}}} \left( {f_{c} \pm \frac{{{3}\alpha }}{{4}}} \right)} \right|{ = }\left| {S_{{{\text{QPSK}}}}^{{{{1} \mathord{\left/ {\vphantom {{1} T}} \right. \kern-0pt} T}}} \left( {f_{c} \pm \frac{{{3}\alpha }}{{4}}} \right)} \right| \approx \frac{E}{4T}\left| {Q\left( {\frac{{5}}{{{4}T}}} \right)} \right|\left| {Q\left( {\frac{{1}}{{{4}T}}} \right)} \right| \approx \frac{{{2}TE}}{{5{\uppi }^{2} }}$, OQPSK signal does not have theoretical value at this cyclic frequency.
5. When $$\alpha = \frac{{1}}{T}$$, $$f{ = }f_{c} \pm \alpha$$.
  $\left| {S_{{{\text{BPSK}}}}^{{{{1} \mathord{\left/ {\vphantom {{1} T}} \right. \kern-0pt} T}}} \left( {f_{c} \pm \alpha } \right)} \right|{ = }\left| {S_{{{\text{QPSK}}}}^{{{{1} \mathord{\left/ {\vphantom {{1} T}} \right. \kern-0pt} T}}} \left( {f_{c} \pm \alpha } \right)} \right| \approx \frac{E}{4T}\left| {Q\left( {\frac{{3}}{{{2}T}}} \right)} \right|\left| {Q\left( {\frac{{1}}{{{2}T}}} \right)} \right| \approx \frac{TE}{{{{3\uppi }}^{2} }}$, OQPSK signal does not have theoretical value at this cyclic frequency.
6. When $$\alpha = \frac{{2}}{T}$$, $$f{ = }f_{c}$$
  $$ \left| {S_{{{\text{BPSK}}}}^{{{{2} \mathord{\left/ {\vphantom {{2} T}} \right. \kern-0pt} T}}} \left( {f_{c} } \right)} \right|{ = }\left| {S_{{{\text{QPSK}}}}^{{{{2} \mathord{\left/ {\vphantom {{2} T}} \right. \kern-0pt} T}}} \left( {f_{c} } \right)} \right|{ = }\left| {S_{{{\text{OQPSK}}}}^{{{{2} \mathord{\left/ {\vphantom {{2} T}} \right. \kern-0pt} T}}} \left( {f_{c} } \right)} \right| \approx \frac{E}{4T}\left| {Q\left( \frac{1}{T} \right)} \right|^{{2}} \approx {0} $$
7. When $$\alpha = \frac{{2}}{T}$$, $$f{ = }f_{c} \pm \frac{\alpha }{{4}}$$
  $$ \left| {S_{{{\text{BPSK}}}}^{{{{2} \mathord{\left/ {\vphantom {{2} T}} \right. \kern-0pt} T}}} \left( {f_{c} \pm \frac{\alpha }{{4}}} \right)} \right|{ = }\left| {S_{{{\text{QPSK}}}}^{{{{2} \mathord{\left/ {\vphantom {{2} T}} \right. \kern-0pt} T}}} \left( {f_{c} \pm \frac{\alpha }{{4}}} \right)} \right|{ = }\left| {S_{{{\text{OQPSK}}}}^{{{{2} \mathord{\left/ {\vphantom {{2} T}} \right. \kern-0pt} T}}} \left( {f_{c} \pm \frac{\alpha }{{4}}} \right)} \right| \approx \frac{E}{4T}\left| {Q\left( {\frac{{3}}{{{2}T}}} \right)} \right|\left| {Q\left( {\frac{{1}}{{{2}T}}} \right)} \right| \approx \frac{TE}{{{{3\uppi  }}^{2} }} $$
8. When $$\alpha = \frac{{2}}{T}$$, $$f{ = }f_{c} \pm \frac{\alpha }{{2}}$$
  $$ \left| {S_{{{\text{BPSK}}}}^{{{{2} \mathord{\left/ {\vphantom {{2} T}} \right. \kern-0pt} T}}} \left( {f_{c} \pm \frac{\alpha }{{2}}} \right)} \right|{ = }\left| {S_{{{\text{QPSK}}}}^{{{{2} \mathord{\left/ {\vphantom {{2} T}} \right. \kern-0pt} T}}} \left( {f_{c} \pm \frac{\alpha }{{2}}} \right)} \right|{ = }\left| {S_{{{\text{OQPSK}}}}^{{{{2} \mathord{\left/ {\vphantom {{2} T}} \right. \kern-0pt} T}}} \left( {f_{c} \pm \frac{\alpha }{{2}}} \right)} \right| \approx \frac{E}{4T}\left| {Q\left( {\frac{{2}}{T}} \right)} \right|\left| {Q\left( {0} \right)} \right| \approx {0} $$
9. When $$\alpha = \frac{{2}}{T}$$, $$f{ = }f_{c} \pm \frac{{{3}\alpha }}{{4}}$$
  $$ \left| {S_{{{\text{BPSK}}}}^{{{{2} \mathord{\left/ {\vphantom {{2} T}} \right. \kern-0pt} T}}} \left( {f_{c} \pm \frac{{{3}\alpha }}{{4}}} \right)} \right|{ = }\left| {S_{{{\text{QPSK}}}}^{{{{2} \mathord{\left/ {\vphantom {{2} T}} \right. \kern-0pt} T}}} \left( {f_{c} \pm \frac{{{3}\alpha }}{{4}}} \right)} \right|{ = }\left| {S_{{{\text{OQPSK}}}}^{{{{2} \mathord{\left/ {\vphantom {{2} T}} \right. \kern-0pt} T}}} \left( {f_{c} \pm \frac{{{3}\alpha }}{{4}}} \right)} \right| \approx \frac{E}{4T}\left| {Q\left( {\frac{{5}}{{{2}T}}} \right)} \right|\left| {Q\left( {\frac{{1}}{{{2}T}}} \right)} \right| \approx \frac{TE}{{{{10\uppi  }}^{2} }} $$
10. When $$\alpha = \frac{{2}}{T}$$, $$f{ = }f_{c} \pm \alpha$$
  $$ \left| {S_{{{\text{BPSK}}}}^{{{{2} \mathord{\left/ {\vphantom {{2} T}} \right. \kern-0pt} T}}} \left( {f_{c} \pm \alpha } \right)} \right|{ = }\left| {S_{{{\text{QPSK}}}}^{{{{2} \mathord{\left/ {\vphantom {{2} T}} \right. \kern-0pt} T}}} \left( {f_{c} \pm \alpha } \right)} \right|{ = }\left| {S_{{{\text{OQPSK}}}}^{{{{2} \mathord{\left/ {\vphantom {{2} T}} \right. \kern-0pt} T}}} \left( {f_{c} \pm \alpha } \right)} \right| \approx \frac{E}{4T}\left| {Q\left( {\frac{{3}}{T}} \right)} \right|\left| {Q\left( {\frac{{1}}{T}} \right)} \right| \approx {0} $$
11. When $$\alpha = \frac{{3}}{T}$$, $$f{ = }f_{c}$$.
  $\left| {S_{{{\text{BPSK}}}}^{{{{3} \mathord{\left/ {\vphantom {{3} T}} \right. \kern-0pt} T}}} \left( {f_{c} } \right)} \right|{ = }\left| {S_{{{\text{QPSK}}}}^{{{{3} \mathord{\left/ {\vphantom {{3} T}} \right. \kern-0pt} T}}} \left( {f_{c} } \right)} \right| \approx \frac{E}{4T}\left| {Q\left( {\frac{3}{{{2}T}}} \right)} \right|^{{2}} \approx \frac{TE}{{{{9\uppi }}^{2} }}$, OQPSK signal does not have theoretical value at this cyclic frequency.
12. When $$\alpha = \frac{{3}}{T}$$, $$f{ = }f_{c} \pm \frac{\alpha }{{4}}$$.
  $\left| {S_{{{\text{BPSK}}}}^{{{{3} \mathord{\left/ {\vphantom {{3} T}} \right. \kern-0pt} T}}} \left( {f_{c} \pm \frac{\alpha }{{4}}} \right)} \right|{ = }\left| {S_{{{\text{QPSK}}}}^{{{{3} \mathord{\left/ {\vphantom {{3} T}} \right. \kern-0pt} T}}} \left( {f_{c} \pm \frac{\alpha }{{4}}} \right)} \right| \approx \frac{E}{4T}\left| {Q\left( {\frac{{9}}{{{4}T}}} \right)} \right|\left| {Q\left( {\frac{3}{{{4}T}}} \right)} \right| \approx \frac{{{2}TE}}{{{{27\uppi  }}^{2} }}$, OQPSK signal does not have theoretical value at this cyclic frequency.
13. When $$\alpha = \frac{{3}}{T}$$, $$f{ = }f_{c} \pm \frac{\alpha }{{2}}$$.
  $\left| {S_{{{\text{BPSK}}}}^{{{{3} \mathord{\left/ {\vphantom {{3} T}} \right. \kern-0pt} T}}} \left( {f_{c} \pm \frac{\alpha }{{2}}} \right)} \right|{ = }\left| {S_{{{\text{QPSK}}}}^{{{{3} \mathord{\left/ {\vphantom {{3} T}} \right. \kern-0pt} T}}} \left( {f_{c} \pm \frac{\alpha }{{2}}} \right)} \right| \approx \frac{E}{4T}\left| {Q\left( {\frac{{3}}{{{2}T}}} \right)} \right| \approx {0}$, OQPSK signal does not have theoretical value at this cyclic frequency.
14. When $$\alpha = \frac{{3}}{T}$$, $$f{ = }f_{c} \pm \frac{{{3}\alpha }}{{4}}$$.
  $\left| {S_{{{\text{BPSK}}}}^{{{{3} \mathord{\left/ {\vphantom {{3} T}} \right. \kern-0pt} T}}} \left( {f_{c} \pm \frac{{{3}\alpha }}{{4}}} \right)} \right|{ = }\left| {S_{{{\text{QPSK}}}}^{{{{3} \mathord{\left/ {\vphantom {{3} T}} \right. \kern-0pt} T}}} \left( {f_{c} \pm \frac{{{3}\alpha }}{{4}}} \right)} \right| \approx \frac{E}{4T}\left| {Q\left( {\frac{{{15}}}{{{4}T}}} \right)} \right|\left| {Q\left( {\frac{{3}}{{{4}T}}} \right)} \right| \approx \frac{{{2}TE}}{{45{\uppi }^{2} }}$, OQPSK signal does not have theoretical value at this cyclic frequency.
15. When $$\alpha = \frac{{3}}{T}$$, $$f{ = }f_{c} \pm \alpha$$.
  $\left| {S_{{{\text{BPSK}}}}^{{{{3} \mathord{\left/ {\vphantom {{3} T}} \right. \kern-0pt} T}}} \left( {f_{c} \pm \alpha } \right)} \right|{ = }\left| {S_{{{\text{QPSK}}}}^{{{{3} \mathord{\left/ {\vphantom {{3} T}} \right. \kern-0pt} T}}} \left( {f_{c} \pm \alpha } \right)} \right| \approx \frac{E}{4T}\left| {Q\left( {\frac{{9}}{{{2}T}}} \right)} \right|\left| {Q\left( {\frac{{3}}{{{2}T}}} \right)} \right| \approx \frac{TE}{{{{27\uppi  }}^{2} }}$, OQPSK signal does not have theoretical value at this cyclic frequency.
16. When $$\alpha \ge \frac{{4}}{T}$$.
  $\left| {S_{{{\text{BPSK}}}}^{\alpha } \left( f \right)} \right|{ = }\left| {S_{{{\text{QPSK}}}}^{\alpha } \left( f \right)} \right| \ll \frac{TE}{{{\uppi }^{2} }}$, this case can be ignored, OQPSK signal does not have theoretical value at this cyclic frequency.

From the above calculation, when $\alpha = \frac{{1}}{T}$ or $\frac{{3}}{T}$, OQPSK signal does not have theoretical value at this cyclic frequency. However, BPSK and QPSK signals have the maximum spectral value in section $f{ = }f_{c}$. In the $\left( {f_{c} {,}f_{c} \pm \frac{\alpha }{2}} \right]$ interval, the spectrum value decreases monotonically from the maximum value to 0. In the $\left( {f_{c} { + }\frac{\alpha }{{2}},{ }f_{c} { + }\alpha } \right]$ and $\left( {f_{c} - \frac{\alpha }{{2}}{,}{ }f_{c} - \alpha } \right]$ intervals, the spectral value presents a parabolic curve distribution, increasing first and then decreasing. When $\alpha = \frac{{2}}{T}$, the distribution of the three signals in the $\left[ {f_{c} {,}{ }f_{c} \pm \frac{\alpha }{2}} \right]$, $\left[ {f_{c} { + }\frac{\alpha }{{2}}{,}{ }f_{c} { + }\alpha } \right]$ and $\left[ {f_{c} { - }\frac{\alpha }{{2}}{,}{ }f_{c} { - }\alpha } \right]$ intervals is parabolic, and the spectrum values of $f\;{ = }\;f_{c}$, $f\;{ = }\;f_{c} \pm \frac{\alpha }{{2}}$ and $f{ = }f_{c} \pm \alpha$ are 0. When $\alpha \ge \frac{{4}}{T}$, the spectrum values of the three types of signals are ignored.

(2) When $\alpha = \pm {2}f_{c} { + }\frac{n}{T}$, the effective value of the cyclic spectrum is mainly distributed near $f{ = 0}$. Similar to $\alpha = \frac{n}{T}$, only $\left( {\alpha \;{ > }\;0{,}\;f\; \ge \;0} \right)$ region is analyzed here, and other regions also have similar characteristics.

1. When $$\alpha = {2}f_{c}$$, $$f \ge {0}$$.
  $\left| {S_{{{\text{BPSK}}}}^{{{2}f_{c} }} \left( f \right)} \right| \approx \frac{E}{{{4}T}}\left| {Q\left( f \right)} \right|^{2}$, QPSK signal and OQPSK signal have no theoretical value at this cyclic frequency.
  When $f \ge {0}$, function $Q\left( f \right)$ decreases monotonically, so $\left| {S_{{{\text{BPSK}}}}^{{{2}f_{c} }} \left( f \right)} \right|$ has a maximum value at $f{ = 0}$, that is, $\left| {S_{{{\text{BPSK}}}}^{{{2}f_{c} }} \left( {0} \right)} \right| \approx \frac{TE}{{4}}$, and decreases to 0 with the gradual increase of *f*.
2. When $$\alpha = {2}f_{c} \pm \frac{{1}}{T}$$, $$f{ = 0}$$.
  $\left| {S_{{{\text{BPSK}}}}^{{{2}f_{c} \pm {{1} \mathord{\left/ {\vphantom {{1} T}} \right. \kern-0pt} T}}} \left( 0 \right)} \right|{ = }\left| {S_{{{\text{OQPSK}}}}^{{{2}f_{c} \pm {{1} \mathord{\left/ {\vphantom {{1} T}} \right. \kern-0pt} T}}} \left( 0 \right)} \right| \approx \frac{E}{{{4}T}}\left| {Q\left( {\frac{1}{{{2}T}}} \right)} \right|^{2} \approx \frac{TE}{{{\uppi }^{2} }}$, QPSK signal does not have theoretical value at this cyclic frequency.
3. When $$\alpha = {2}f_{c} \pm \frac{{1}}{T}$$, $$f{ = }\frac{{1}}{{{4}T}}$$.
  $\left| {S_{{{\text{BPSK}}}}^{{{2}f_{c} \pm {{1} \mathord{\left/ {\vphantom {{1} T}} \right. \kern-0pt} T}}} \left( {{1 \mathord{\left/ {\vphantom {1 {{4}T}}} \right. \kern-0pt} {{4}T}}} \right)} \right|{ = }\left| {S_{{{\text{OQPSK}}}}^{{{2}f_{c} \pm {{1} \mathord{\left/ {\vphantom {{1} T}} \right. \kern-0pt} T}}} \left( {{1 \mathord{\left/ {\vphantom {1 {{4}T}}} \right. \kern-0pt} {{4}T}}} \right)} \right| \approx \frac{E}{4T}\left| {Q\left( \frac{1}{4T} \right)} \right|\left| {Q\left( \frac{3}{4T} \right)} \right| \approx \frac{{{2}TE}}{{3\pi^{2} }}$, QPSK signal does not have theoretical value at this cyclic frequency.
4. When $$\alpha = {2}f_{c} \pm \frac{{1}}{T}$$, $$f{ = }\frac{{1}}{{{2}T}}$$.
  $\left| {S_{{{\text{BPSK}}}}^{{{2}f_{c} \pm {{1} \mathord{\left/ {\vphantom {{1} T}} \right. \kern-0pt} T}}} \left( {{{1} \mathord{\left/ {\vphantom {{1} {{2}T}}} \right. \kern-0pt} {{2}T}}} \right)} \right|{ = }\left| {S_{{{\text{OQPSK}}}}^{{{2}f_{c} \pm {{1} \mathord{\left/ {\vphantom {{1} T}} \right. \kern-0pt} T}}} \left( {{{1} \mathord{\left/ {\vphantom {{1} {{2}T}}} \right. \kern-0pt} {{2}T}}} \right)} \right| \approx \frac{E}{{{4}T}}\left| {Q\left( {0} \right)} \right|\left| {Q\left( \frac{1}{T} \right)} \right| \approx {0}$, QPSK signal does not have theoretical value at this cyclic frequency.
5. When $$\alpha = {2}f_{c} \pm \frac{{1}}{T}$$, $$f{ = }\frac{{3}}{{{4}T}}$$.
  $\left| {S_{{{\text{BPSK}}}}^{{{2}f_{c} \pm {{1} \mathord{\left/ {\vphantom {{1} T}} \right. \kern-0pt} T}}} \left( {{{3} \mathord{\left/ {\vphantom {{3} {{4}T}}} \right. \kern-0pt} {{4}T}}} \right)} \right|{ = }\left| {S_{{{\text{OQPSK}}}}^{{{2}f_{c} \pm {{1} \mathord{\left/ {\vphantom {{1} T}} \right. \kern-0pt} T}}} \left( {{{3} \mathord{\left/ {\vphantom {{3} {{4}T}}} \right. \kern-0pt} {{4}T}}} \right)} \right| \approx \frac{E}{{{4}T}}\left| {Q\left( {\frac{1}{{{4}T}}} \right)} \right|\left| {Q\left( {\frac{{5}}{{{4}T}}} \right)} \right| \approx \frac{{{2}TE}}{{{{5\uppi  }}^{2} }}$, QPSK signal does not have theoretical value at this cyclic frequency.
6. When $$\alpha = {2}f_{c} \pm \frac{{1}}{T}$$, $$f{ = }\frac{{1}}{T}$$.
  $\left| {S_{{{\text{BPSK}}}}^{{{2}f_{c} \pm {{1} \mathord{\left/ {\vphantom {{1} T}} \right. \kern-0pt} T}}} \left( {{{1} \mathord{\left/ {\vphantom {{1} T}} \right. \kern-0pt} T}} \right)} \right|{ = }\left| {S_{{{\text{OQPSK}}}}^{{{2}f_{c} \pm {{1} \mathord{\left/ {\vphantom {{1} T}} \right. \kern-0pt} T}}} \left( {{{1} \mathord{\left/ {\vphantom {{1} T}} \right. \kern-0pt} T}} \right)} \right| \approx \frac{E}{{{4}T}}\left| {Q\left( {\frac{1}{{{2}T}}} \right)} \right|\left| {Q\left( {\frac{{3}}{{{2}T}}} \right)} \right| \approx \frac{TE}{{{{3\uppi  }}^{2} }}$, OQPSK signal does not have theoretical value at this cyclic frequency.
7. When $$\alpha = {2}f_{c} \pm \frac{{2}}{T}$$, $$f{ = 0}$$.
  $\left| {S_{{{\text{BPSK}}}}^{{{2}f_{c} \pm {{2} \mathord{\left/ {\vphantom {{2} T}} \right. \kern-0pt} T}}} \left( 0 \right)} \right| \approx \frac{E}{{{4}T}}\left| {Q\left( \frac{1}{T} \right)} \right|^{2} \approx 0$, QPSK signal and OQPSK signal have no theoretical value at this cyclic frequency.
8. When $$\alpha = {2}f_{c} \pm \frac{{2}}{T}$$, $$f{ = }\frac{{1}}{{{4}T}}$$.
  $\left| {S_{{{\text{BPSK}}}}^{{{2}f_{c} \pm {{2} \mathord{\left/ {\vphantom {{2} T}} \right. \kern-0pt} T}}} \left( {{{1} \mathord{\left/ {\vphantom {{1} {{4}T}}} \right. \kern-0pt} {{4}T}}} \right)} \right| \approx \frac{E}{{{4}T}}\left| {Q\left( \frac{3}{4T} \right)} \right|\left| {Q\left( \frac{5}{4T} \right)} \right| \approx \frac{2TE}{{15{\uppi }^{2} }}$, QPSK signal and OQPSK signal have no theoretical value at this cyclic frequency.
9. When $$\alpha = {2}f_{c} \pm \frac{{2}}{T}$$, $$f{ = }\frac{{1}}{{{2}T}}$$.
  $\left| {S_{{{\text{BPSK}}}}^{{{2}f_{c} \pm {{2} \mathord{\left/ {\vphantom {{2} T}} \right. \kern-0pt} T}}} \left( {{{1} \mathord{\left/ {\vphantom {{1} {{2}T}}} \right. \kern-0pt} {{2}T}}} \right)} \right| \approx \frac{E}{{{4}T}}\left| {Q\left( {\frac{{1}}{{{2}T}}} \right)} \right|\left| {Q\left( {\frac{{3}}{{{2}T}}} \right)} \right| \approx \frac{2TE}{{{{3\uppi }}^{2} }}$, QPSK signal and OQPSK signal have no theoretical value at this cyclic frequency.
10. When $$\alpha = {2}f_{c} \pm \frac{{2}}{T}$$, $$f{ = }\frac{{3}}{{{4}T}}$$.
  $\left| {S_{{{\text{BPSK}}}}^{{{2}f_{c} \pm {{2} \mathord{\left/ {\vphantom {{2} T}} \right. \kern-0pt} T}}} \left( {{{3} \mathord{\left/ {\vphantom {{3} {{4}T}}} \right. \kern-0pt} {{4}T}}} \right)} \right| \approx \frac{E}{{{4}T}}\left| {Q\left( {\frac{{1}}{{{4}T}}} \right)} \right|\left| {Q\left( {\frac{{7}}{{{4}T}}} \right)} \right| \approx \frac{{{2}TE}}{{{{ 7\uppi }}^{2} }}$, QPSK signal and OQPSK signal have no theoretical value at this cyclic frequency.
11. When $$\alpha = {2}f_{c} \pm \frac{{2}}{T}$$, $$f{ = }\frac{{1}}{T}$$.
  $\left| {S_{{{\text{BPSK}}}}^{{{2}f_{c} \pm {{2} \mathord{\left/ {\vphantom {{2} T}} \right. \kern-0pt} T}}} \left( {{{1} \mathord{\left/ {\vphantom {{1} T}} \right. \kern-0pt} T}} \right)} \right| \approx \frac{E}{{{4}T}}\left| {Q\left( {0} \right)} \right|\left| {Q\left( {\frac{{2}}{T}} \right)} \right| \approx {0}$, QPSK signal and OQPSK signal have no theoretical value at this cyclic frequency.
12. When $$\alpha = {2}f_{c} \pm \frac{{3}}{T}$$, $$f{ = 0}$$.
  $\left| {S_{{{\text{BPSK}}}}^{{{2}f_{c} \pm {{3} \mathord{\left/ {\vphantom {{3} T}} \right. \kern-0pt} T}}} \left( {0} \right)} \right|{ = }\left| {S_{{{\text{OQPSK}}}}^{{{2}f_{c} \pm {{3} \mathord{\left/ {\vphantom {{3} T}} \right. \kern-0pt} T}}} \left( {0} \right)} \right| \approx \frac{E}{{{4}T}}\left| {Q\left( {\frac{{3}}{{{2}T}}} \right)} \right|^{{2}} \approx \frac{TE}{{{{9\uppi }}^{2} }}$, QPSK signal does not have theoretical value at this cyclic frequency.
13. When $$\alpha = {2}f_{c} \pm \frac{{3}}{T}$$, $$f{ = }\frac{{1}}{{{4}T}}$$.
  $\left| {S_{{{\text{BPSK}}}}^{{{2}f_{c} \pm {{3} \mathord{\left/ {\vphantom {{3} T}} \right. \kern-0pt} T}}} \left( {{{1} \mathord{\left/ {\vphantom {{1} {{4}T}}} \right. \kern-0pt} {{4}T}}} \right)} \right|{ = }\left| {S_{{{\text{OQPSK}}}}^{{{2}f_{c} \pm {{3} \mathord{\left/ {\vphantom {{3} T}} \right. \kern-0pt} T}}} \left( {{{1} \mathord{\left/ {\vphantom {{1} {{4}T}}} \right. \kern-0pt} {{4}T}}} \right)} \right| \approx \frac{E}{{{4}T}}\left| {Q\left( {\frac{{7}}{{{4}T}}} \right)} \right|\left| {Q\left( {\frac{{5}}{{{4}T}}} \right)} \right| \approx \frac{2TE}{{{{35\uppi }}^{2} }}$, QPSK signal does not have theoretical value at this cyclic frequency.
14. When $$\alpha = {2}f_{c} \pm \frac{{3}}{T}$$, $$f{ = }\frac{1}{2T}$$.
  $\left| {S_{{{\text{BPSK}}}}^{{{2}f_{c} \pm {{3} \mathord{\left/ {\vphantom {{3} T}} \right. \kern-0pt} T}}} \left( {{{1} \mathord{\left/ {\vphantom {{1} {{2}T}}} \right. \kern-0pt} {{2}T}}} \right)} \right|{ = }\left| {S_{{{\text{OQPSK}}}}^{{{2}f_{c} \pm {{3} \mathord{\left/ {\vphantom {{3} T}} \right. \kern-0pt} T}}} \left( {{{1} \mathord{\left/ {\vphantom {{1} {{2}T}}} \right. \kern-0pt} {{2}T}}} \right)} \right| \approx \frac{E}{{{4}T}}\left| {Q\left( {\frac{{2}}{T}} \right)} \right|\left| {Q\left( {\frac{{1}}{T}} \right)} \right| \approx {0}$, QPSK signal does not have theoretical value at this cyclic frequency.
15. When $$\alpha = {2}f_{c} \pm \frac{{3}}{T}$$, $$f{ = }\frac{3}{{{4}T}}$$.
  $\left| {S_{{{\text{BPSK}}}}^{{{2}f_{c} \pm {{3} \mathord{\left/ {\vphantom {{3} T}} \right. \kern-0pt} T}}} \left( {{{3} \mathord{\left/ {\vphantom {{3} {{4}T}}} \right. \kern-0pt} {{4}T}}} \right)} \right|{ = }\left| {S_{{{\text{OQPSK}}}}^{{{2}f_{c} \pm {{3} \mathord{\left/ {\vphantom {{3} T}} \right. \kern-0pt} T}}} \left( {{{3} \mathord{\left/ {\vphantom {{3} {{4}T}}} \right. \kern-0pt} {{4}T}}} \right)} \right| \approx \frac{E}{{{4}T}}\left| {Q\left( {\frac{{9}}{4T}} \right)} \right|\left| {Q\left( {\frac{{3}}{4T}} \right)} \right| \approx \frac{2TE}{{{{27\uppi }}^{2} }}$, QPSK signal does not have theoretical value at this cyclic frequency.
16. When $$\alpha = {2}f_{c} \pm \frac{{3}}{T}$$, $$f{ = }\frac{{1}}{T}$$.
  $\left| {S_{{{\text{BPSK}}}}^{{{2}f_{c} \pm {{3} \mathord{\left/ {\vphantom {{3} T}} \right. \kern-0pt} T}}} \left( {{{1} \mathord{\left/ {\vphantom {{1} T}} \right. \kern-0pt} T}} \right)} \right|{ = }\left| {S_{{{\text{OQPSK}}}}^{{{2}f_{c} \pm {{3} \mathord{\left/ {\vphantom {{3} T}} \right. \kern-0pt} T}}} \left( {{{1} \mathord{\left/ {\vphantom {{1} T}} \right. \kern-0pt} T}} \right)} \right| \approx \frac{E}{{{4}T}}\left| {Q\left( {\frac{{5}}{{{2}T}}} \right)} \right|\left| {Q\left( {\frac{{1}}{{{2}T}}} \right)} \right| \approx \frac{TE}{{{{5\uppi }}^{2} }}$, QPSK signal does not have theoretical value at this cyclic frequency.
17. When $$\alpha \ge {2}f_{c} { + }\frac{{4}}{T}$$ or $$\alpha \le {2}f_{c} { - }\frac{{4}}{T}$$.
  $\left| {S_{{{\text{BPSK}}}}^{\alpha } \left( f \right)} \right|{ = }\left| {S_{{{\text{OQPSK}}}}^{\alpha } \left( f \right)} \right| \ll \frac{TE}{{{\uppi }^{2} }}$, this case can be ignored, and QPSK signal does not have theoretical value at this cyclic frequency.

According to the above analysis, there is no effective cyclic spectrum value of QPSK signal in the whole section of $\alpha = {2}f_{c} \pm \frac{n}{T}$ (*n* is an integer). In section $\alpha = {2}f_{c}$, when $f \ge {0}$, the OQPSK signal does not have an effective cyclic spectrum value, and the cyclic spectrum value of BPSK signal decreases monotonically. In section $\alpha = {2}f_{c} \pm \frac{{1}}{T}$ and $\alpha = {2}f_{c} \pm \frac{{3}}{T}$, when interval $f \in \left[ {0{,}{{{ }1} \mathord{\left/ {\vphantom {{{ }1} T}} \right. \kern-0pt} T}} \right]$, the cyclic spectral values of BPSK and OQPSK signals show an inverse parabola distribution that decreases first and then increases. In section $\alpha = {2}f_{c} \pm \frac{{2}}{T}$ and interval $f \in \left[ {0{,}{{{ }1} \mathord{\left/ {\vphantom {{{ }1} T}} \right. \kern-0pt} T}} \right]$, there is no effective cyclic spectrum of OQPSK signals, and the cyclic spectrum of BPSK signals increases first and then decreases in parabolic distribution. When $\alpha \ge {2}f_{c} { + }\frac{{4}}{T}$ or $\alpha \le {2}f_{c} - \frac{{4}}{T}$, the cyclic spectrum values of BPSK signal and OQPSK signal are small and ignored.

### Cyclic spectrum characteristics of mixed double-signal

**Figure 4:**
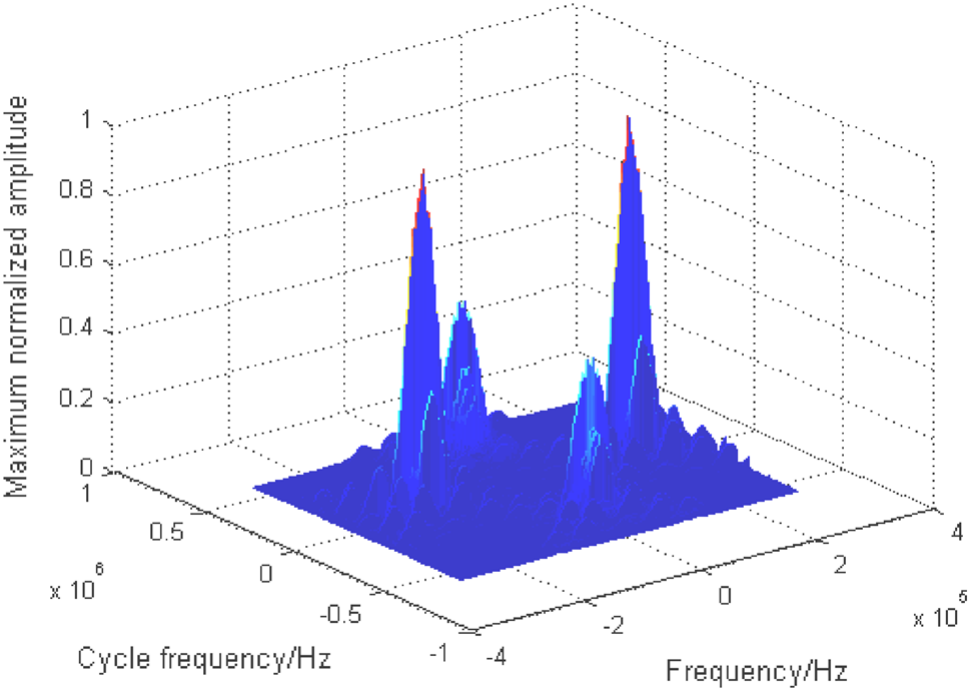
Cyclic spectrum of BPSK + QPSK.

**Figure 5:**
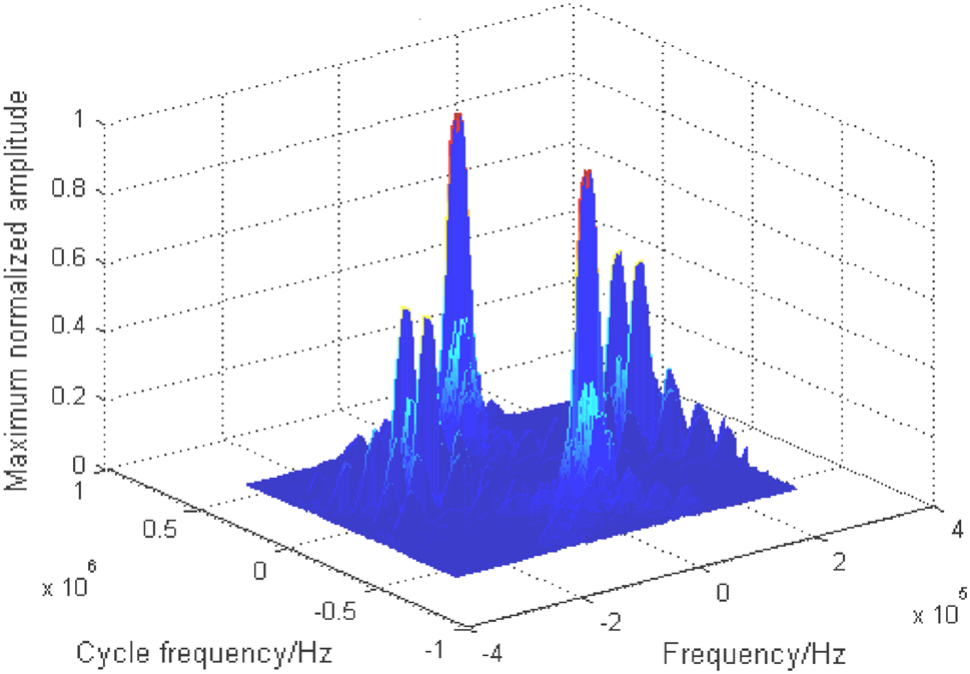
Cyclic spectrum ($\alpha \ne 0$) of BPSK + QPSK.

Due to space constraints, BPSK + QPSK is taken as an example to analyze the cyclic spectrum characteristics of mixed signals. The cyclic spectrum equation of BPSK + QPSK is as follows:

5 $$ S_{{\text{BPSK + QPSK}}}^{\alpha } \left( f \right) = \left\{ {\begin{array}{*{20}c} \begin{gathered} \frac{{E_{b} }}{{4T_{b} }}\left[ {Q_{b} \left( {f - f_{cb} + \frac{\alpha }{2}} \right)Q_{b}^{ * } \left( {f - f_{cb} - \frac{\alpha }{2}} \right) + Q_{b} \left( {f + f_{cb} + \frac{\alpha }{2}} \right)Q_{b}^{ * } \left( {f + f_{cb} - \frac{\alpha }{2}} \right)} \right]\exp \left( { - {{j2\uppi }}\alpha t_{0b} } \right){, } \;\; {\text{when}}{ }\alpha = \frac{n}{{T_{b} }} \hfill \\ \frac{{E_{q} }}{{4T_{q} }}\left[ {Q_{q} \left( {f - f_{cq} + \frac{\alpha }{2}} \right)Q_{q}^{ * } \left( {f - f_{cq} - \frac{\alpha }{2}} \right) + Q_{q} \left( {f + f_{cq} + \frac{\alpha }{2}} \right)Q_{q}^{ * } \left( {f + f_{cq} - \frac{\alpha }{2}} \right)} \right]\exp \left( { - {{j2\uppi }}\alpha t_{0q} } \right), \;\; {\text{when}}{ }\alpha = \frac{n}{{T_{q} }} \hfill \\ \end{gathered} \\ \begin{gathered} \frac{{E_{b} }}{{4T_{b} }}\left[ {\exp \left( {j2\phi_{0b} } \right)Q_{b} \left( {f - f_{cb} + \frac{\alpha }{2}} \right)Q_{b}^{ * } \left( {f + f_{cb} - \frac{\alpha }{2}} \right) + {\text{exp}}\left( {{\text{ - j2}}\phi_{{{0}b}} } \right)Q_{b} \left( {f + f_{cb} + \frac{\alpha }{2}} \right)Q_{b}^{ * } \left( {f - f_{cb} - \frac{\alpha }{2}} \right)} \right] \times \hfill \\ \exp \left( { - {{j2\uppi }}\alpha t_{0q} } \right),{ }{\text{when}}{ }\alpha = \pm 2f_{cb} + \frac{n}{{T_{b} }} \hfill \\ \end{gathered} \\ \end{array} } \right. $$

where the variable subscript symbols $b$ and $q$ respectively represent BPSK and QPSK signals. For the mixed signals with the same frequency and rate studied in this paper, $f_{cb} { = }f_{cq}$, $T_{b} { = }T_{q}$, $Q_{b} { = }Q_{q}$. The cyclic spectrum of mixed double-signal is shown in Fig. 4, in which the energy of signals is equal. Similar to the characteristics of the single signal cyclic spectrum, the noise here also has a cross section. The cyclic spectrum values of the signal are suppressed to some extent due to the noise. To avoid the influence of noise, the cyclic spectrum cross section at $\alpha = 0$ is shielded, and the cyclic spectrum characteristics of each signal are highlighted, as shown in Fig. 5.

In the modulation recognition of mixed signals with the same frequency and rate, the energy ratio between signals is a key factor affecting the recognition effect. Therefore, according to the theoretical value of the signal cyclic spectrum in section “Cyclic spectrum characteristics of single signal”, this section makes a theoretical analysis of the characteristics of the cyclic spectrum when the energy ratio between mixed signals is 1:1, 1:4 and 4:1. This part can be regarded as a general analysis of the energy ratio between signals from 1:1 to 1:4 or 1:1 to 4:1. When the energy ratio exceeds 1:4 or 4:1, the signal with high energy is less affected and can be regarded as a single signal, which is beyond the scope of this paper and will not be discussed here. See Table 1 for details, $E_{{{\text{BPSK}}}}$, $E_{{{\text{QPSK}}}}$ and $E_{{{\text{OQPSK}}}}$ respectively represent the energy of BPSK, QPSK and OQPSK signals in mixed signal.

**Table 1:** Theoretical value of cyclic spectrum at different frequency points of mixed double-signal.

Serial number	Frequency point	Type	BPSK + QPSK		BPSK + OQPSK		QPSK + OQPSK	
1	$\alpha = \frac{{1}}{T}$ $f{ = }f_{c}$	Theoretical value	${{TE_{{{\text{BPSK}}}} } \mathord{\left/ {\vphantom {{TE_{{{\text{BPSK}}}} } {{\uppi }^{2} }}} \right. \kern-0pt} {{\uppi }^{2} }}{ + }{{TE_{{{\text{QPSK}}}} } \mathord{\left/ {\vphantom {{TE_{{{\text{QPSK}}}} } {{\uppi }^{2} }}} \right. \kern-0pt} {{\uppi }^{2} }}$		${{TE_{{{\text{BPSK}}}} } \mathord{\left/ {\vphantom {{TE_{{{\text{BPSK}}}} } {{\uppi }^{2} }}} \right. \kern-0pt} {{\uppi }^{2} }}$		${{TE_{{{\text{QPSK}}}} } \mathord{\left/ {\vphantom {{TE_{{{\text{QPSK}}}} } {{\uppi }^{2} }}} \right. \kern-0pt} {{\uppi }^{2} }}$	
		Maximum normalized spectral value	$E_{{{\text{BPSK}}}} {:}E_{{{\text{QPSK}}}}$	/	$E_{{{\text{BPSK}}}} {:}E_{{{\text{OQPSK}}}}$	/	$E_{{{\text{QPSK}}}} {:}E_{{{\text{OQPSK}}}}$	/
			1:1	0.81	1:1	0.41	1:1	1.00
			1:4	1.00	1:4	0.20	1:4	0.25
			4:1	0.51	4:1	0.41	4:1	1.00
2	$\alpha = \frac{{1}}{T}$ $f{ = }f_{c} \pm \frac{\alpha }{{4}}$	Theoretical value	${{{2}TE_{{{\text{BPSK}}}} } \mathord{\left/ {\vphantom {{{2}TE_{{{\text{BPSK}}}} } {{{3\uppi }}^{2} }}} \right. \kern-0pt} {{{3\uppi }}^{2} }}{ + }{{{2}TE_{{{\text{QPSK}}}} } \mathord{\left/ {\vphantom {{{2}TE_{{{\text{QPSK}}}} } {{{3\uppi }}^{2} }}} \right. \kern-0pt} {{{3\uppi }}^{2} }}$		${{{2}TE_{{{\text{BPSK}}}} } \mathord{\left/ {\vphantom {{{2}TE_{{{\text{BPSK}}}} } {{{3\uppi }}^{2} }}} \right. \kern-0pt} {{{3\uppi }}^{2} }}$		${{{2}TE_{{{\text{QPSK}}}} } \mathord{\left/ {\vphantom {{{2}TE_{{{\text{QPSK}}}} } {{{3\uppi }}^{2} }}} \right. \kern-0pt} {{{3\uppi }}^{2} }}$	
		Maximum normalized spectral value	$E_{{{\text{BPSK}}}} {:}E_{{{\text{QPSK}}}}$	/	$E_{{{\text{BPSK}}}} {:}E_{{{\text{OQPSK}}}}$	/	$E_{{{\text{QPSK}}}} {:}E_{{{\text{OQPSK}}}}$	/
			1:1	0.54	1:1	0.27	1:1	0.67
			1:4	0.67	1:4	0.13	1:4	0.17
			4:1	0.34	4:1	0.27	4:1	0.67
3	$\alpha = \frac{{1}}{T}$ $f{ = }f_{c} \pm \frac{\alpha }{{2}}$	Theoretical value	0		0		0	
		Maximum normalized spectral value	$E_{{{\text{BPSK}}}} {:}E_{{{\text{QPSK}}}}$	/	$E_{{{\text{BPSK}}}} {:}E_{{{\text{OQPSK}}}}$	/	$E_{{{\text{QPSK}}}} {:}E_{{{\text{OQPSK}}}}$	/
			1:1	0	1:1	0	1:1	0
			1:4	0	1:4	0	1:4	0
			4:1	0	4:1	0	4:1	0
4	$\alpha = \frac{{1}}{T}$ $f{ = }f_{c} \pm \frac{{{3}\alpha }}{{4}}$	Theoretical value	${{{2}TE_{{{\text{BPSK}}}} } \mathord{\left/ {\vphantom {{{2}TE_{{{\text{BPSK}}}} } {{{5\uppi }}^{2} }}} \right. \kern-0pt} {{{5\uppi }}^{2} }}{ + }{{{2}TE_{{{\text{QPSK}}}} } \mathord{\left/ {\vphantom {{{2}TE_{{{\text{QPSK}}}} } {{{5\uppi }}^{2} }}} \right. \kern-0pt} {{{5\uppi }}^{2} }}$		${{{2}TE_{{{\text{BPSK}}}} } \mathord{\left/ {\vphantom {{{2}TE_{{{\text{BPSK}}}} } {{{5\uppi }}^{2} }}} \right. \kern-0pt} {{{5\uppi }}^{2} }}$		${{{2}TE_{{{\text{QPSK}}}} } \mathord{\left/ {\vphantom {{{2}TE_{{{\text{QPSK}}}} } {{{5\uppi }}^{2} }}} \right. \kern-0pt} {{{5\uppi }}^{2} }}$	
		Maximum normalized spectral value	$E_{{{\text{BPSK}}}} {:}E_{{{\text{QPSK}}}}$	/	$E_{{{\text{BPSK}}}} {:}E_{{{\text{OQPSK}}}}$	/	$E_{{{\text{QPSK}}}} {:}E_{{{\text{OQPSK}}}}$	/
			1:1	0.32	1:1	0.16	1:1	0.40
			1:4	0.40	1:4	0.08	1:4	0.10
			4:1	0.20	4:1	0.16	4:1	0.40
5	$\alpha = \frac{{1}}{T}$ $f{ = }f_{c} \pm \alpha$	Theoretical value	${{TE_{{{\text{BPSK}}}} } \mathord{\left/ {\vphantom {{TE_{{{\text{BPSK}}}} } {{{3\uppi }}^{2} }}} \right. \kern-0pt} {{{3\uppi }}^{2} }}{ + }{{TE_{{{\text{QPSK}}}} } \mathord{\left/ {\vphantom {{TE_{{{\text{QPSK}}}} } {{{3\uppi }}^{2} }}} \right. \kern-0pt} {{{3\uppi }}^{2} }}$		${{TE_{{{\text{BPSK}}}} } \mathord{\left/ {\vphantom {{TE_{{{\text{BPSK}}}} } {{{3\uppi }}^{2} }}} \right. \kern-0pt} {{{3\uppi }}^{2} }}$		${{TE_{{{\text{QPSK}}}} } \mathord{\left/ {\vphantom {{TE_{{{\text{QPSK}}}} } {{{3\uppi }}^{2} }}} \right. \kern-0pt} {{{3\uppi }}^{2} }}$	
		Maximum normalized spectral value	$E_{{{\text{BPSK}}}} {:}E_{{{\text{QPSK}}}}$	/	$E_{{{\text{BPSK}}}} {:}E_{{{\text{OQPSK}}}}$	/	$E_{{{\text{QPSK}}}} {:}E_{{{\text{OQPSK}}}}$	/
			1:1	0.27	1:1	0.14	1:1	0.33
			1:4	0.33	1:4	0.07	1:4	0.08
			4:1	0.17	4:1	0.14	4:1	0.33
6	$\alpha = \frac{{2}}{T}$ $f{ = }f_{c}$	Theoretical value	0		0		0	
		Maximum normalized spectral value	$E_{{{\text{BPSK}}}} {:}E_{{{\text{QPSK}}}}$	/	$E_{{{\text{BPSK}}}} {:}E_{{{\text{OQPSK}}}}$	/	$E_{{{\text{QPSK}}}} {:}E_{{{\text{OQPSK}}}}$	/
			1:1	0	1:1	0	1:1	0
			1:4	0	1:4	0	1:4	0
			4:1	0	4:1	0	4:1	0
7	$\alpha = \frac{{2}}{T}$ $f{ = }f_{c} \pm \frac{\alpha }{{4}}$	Theoretical value	${{TE_{{{\text{BPSK}}}} } \mathord{\left/ {\vphantom {{TE_{{{\text{BPSK}}}} } {{{3\uppi }}^{2} }}} \right. \kern-0pt} {{{3\uppi }}^{2} }}{ + }{{TE_{{{\text{QPSK}}}} } \mathord{\left/ {\vphantom {{TE_{{{\text{QPSK}}}} } {{{3\uppi }}^{2} }}} \right. \kern-0pt} {{{3\uppi }}^{2} }}$		${{TE_{{{\text{BPSK}}}} } \mathord{\left/ {\vphantom {{TE_{{{\text{BPSK}}}} } {{{3\uppi }}^{2} }}} \right. \kern-0pt} {{{3\uppi }}^{2} }}{ + }{{TE_{{{\text{OQPSK}}}} } \mathord{\left/ {\vphantom {{TE_{{{\text{OQPSK}}}} } {{{3\uppi }}^{2} }}} \right. \kern-0pt} {{{3\uppi }}^{2} }}$		${{TE_{{{\text{QPSK}}}} } \mathord{\left/ {\vphantom {{TE_{{{\text{QPSK}}}} } {{{3\uppi }}^{2} }}} \right. \kern-0pt} {{{3\uppi }}^{2} }}{ + }{{TE_{{{\text{OQPSK}}}} } \mathord{\left/ {\vphantom {{TE_{{{\text{OQPSK}}}} } {{{3\uppi }}^{2} }}} \right. \kern-0pt} {{{3\uppi }}^{2} }}$	
		Maximum normalized spectral value	$E_{{{\text{BPSK}}}} {:}E_{{{\text{QPSK}}}}$	/	$E_{{{\text{BPSK}}}} {:}E_{{{\text{OQPSK}}}}$	/	$E_{{{\text{QPSK}}}} {:}E_{{{\text{OQPSK}}}}$	/
			1:1	0.27	1:1	0.27	1:1	0.67
			1:4	0.33	1:4	0.33	1:4	0.42
			4:1	0.17	4:1	0.17	4:1	0.42
8	$\alpha = \frac{{2}}{T}$ $f{ = }f_{c} \pm \frac{\alpha }{{2}}$	Theoretical value	0		0		0	
		Maximum normalized spectral value	$E_{{{\text{BPSK}}}} {:}E_{{{\text{QPSK}}}}$	/	$E_{{{\text{BPSK}}}} {:}E_{{{\text{OQPSK}}}}$	/	$E_{{{\text{QPSK}}}} {:}E_{{{\text{OQPSK}}}}$	/
			1:1	0	1:1	0	1:1	0
			1:4	0	1:4	0	1:4	0
			4:1	0	4:1	0	4:1	0
9	$\alpha = \frac{{2}}{T}$ $f{ = }f_{c} \pm \frac{{{3}\alpha }}{{4}}$	Theoretical value	${{TE_{{{\text{BPSK}}}} } \mathord{\left/ {\vphantom {{TE_{{{\text{BPSK}}}} } {{{10\uppi }}^{2} }}} \right. \kern-0pt} {{{10\uppi }}^{2} }}{ + }{{TE_{{{\text{QPSK}}}} } \mathord{\left/ {\vphantom {{TE_{{{\text{QPSK}}}} } {{{10\uppi }}^{2} }}} \right. \kern-0pt} {{{10\uppi }}^{2} }}$		${{TE_{{{\text{BPSK}}}} } \mathord{\left/ {\vphantom {{TE_{{{\text{BPSK}}}} } {{{10\uppi }}^{2} }}} \right. \kern-0pt} {{{10\uppi }}^{2} }}{ + }{{TE_{{{\text{OQPSK}}}} } \mathord{\left/ {\vphantom {{TE_{{{\text{OQPSK}}}} } {{{10\uppi }}^{2} }}} \right. \kern-0pt} {{{10\uppi }}^{2} }}$		${{TE_{{{\text{QPSK}}}} } \mathord{\left/ {\vphantom {{TE_{{{\text{QPSK}}}} } {{{10\uppi }}^{2} }}} \right. \kern-0pt} {{{10\uppi }}^{2} }}{ + }{{TE_{{{\text{OQPSK}}}} } \mathord{\left/ {\vphantom {{TE_{{{\text{OQPSK}}}} } {{{10\uppi }}^{2} }}} \right. \kern-0pt} {{{10\uppi }}^{2} }}$	
		Maximum normalized spectral value	$E_{{{\text{BPSK}}}} {:}E_{{{\text{QPSK}}}}$	/	$E_{{{\text{BPSK}}}} {:}E_{{{\text{OQPSK}}}}$	/	$E_{{{\text{QPSK}}}} {:}E_{{{\text{OQPSK}}}}$	/
			1:1	0.08	1:1	0.08	1:1	0.20
			1:4	0.10	1:4	0.10	1:4	0.13
			4:1	0.05	4:1	0.05	4:1	0.13
10	$\alpha = \frac{{2}}{T}$ $f{ = }f_{c} \pm \alpha$	Theoretical value	0		0		0	
		Maximum normalized spectral value	$E_{{{\text{BPSK}}}} {:}E_{{{\text{QPSK}}}}$	/	$E_{{{\text{BPSK}}}} {:}E_{{{\text{OQPSK}}}}$	/	$E_{{{\text{QPSK}}}} {:}E_{{{\text{OQPSK}}}}$	/
			1:1	0	1:1	0	1:1	0
			1:4	0	1:4	0	1:4	0
			4:1	0	4:1	0	4:1	0
11	$\alpha \ge \frac{{3}}{T}$	Theoretical value	$\ll {{TE} \mathord{\left/ {\vphantom {{TE} {{\uppi }^{2} }}} \right. \kern-0pt} {{\uppi }^{2} }}$		$\ll {{TE} \mathord{\left/ {\vphantom {{TE} {{\uppi }^{2} }}} \right. \kern-0pt} {{\uppi }^{2} }}$		$\ll {{TE} \mathord{\left/ {\vphantom {{TE} {{\uppi }^{2} }}} \right. \kern-0pt} {{\uppi }^{2} }}$	
		Maximum normalized spectral value	$E_{{{\text{BPSK}}}} {:}E_{{{\text{QPSK}}}}$	/	$E_{{{\text{BPSK}}}} {:}E_{{{\text{OQPSK}}}}$	/	$E_{{{\text{QPSK}}}} {:}E_{{{\text{OQPSK}}}}$	/
			1:1	<< 0.1	1:1	<< 0.1	1:1	<< 0.1
			1:4	<< 0.1	1:4	<< 0.1	1:4	<< 0.1
			4:1	<< 0.1	4:1	<< 0.1	4:1	<< 0.1
12	$\alpha = {2}f_{c}$ $f{ = 0}$	Theoretical value	${{TE_{{{\text{BPSK}}}} } \mathord{\left/ {\vphantom {{TE_{{{\text{BPSK}}}} } {4}}} \right. \kern-0pt} {4}}$		${{TE_{{{\text{BPSK}}}} } \mathord{\left/ {\vphantom {{TE_{{{\text{BPSK}}}} } {4}}} \right. \kern-0pt} {4}}$		/	
		Maximum normalized spectral value	$E_{{{\text{BPSK}}}} {:}E_{{{\text{QPSK}}}}$	/	$E_{{{\text{BPSK}}}} {:}E_{{{\text{OQPSK}}}}$	/	$E_{{{\text{QPSK}}}} {:}E_{{{\text{OQPSK}}}}$	/
			1:1	1.00	1:1	1.00	1:1	/
			1:4	0.49	1:4	0.49	1:4	/
			4:1	1.00	4:1	1.00	4:1	/
13	$\alpha = {2}f_{c} \pm \frac{{1}}{T}$ $f{ = 0}$	Theoretical value	${{TE_{{{\text{BPSK}}}} } \mathord{\left/ {\vphantom {{TE_{{{\text{BPSK}}}} } {{\uppi }^{2} }}} \right. \kern-0pt} {{\uppi }^{2} }}$		${{TE_{{{\text{BPSK}}}} } \mathord{\left/ {\vphantom {{TE_{{{\text{BPSK}}}} } {{\uppi }^{2} }}} \right. \kern-0pt} {{\uppi }^{2} }}{ + }{{TE_{{{\text{OQPSK}}}} } \mathord{\left/ {\vphantom {{TE_{{{\text{OQPSK}}}} } {{\uppi }^{2} }}} \right. \kern-0pt} {{\uppi }^{2} }}$		${{TE_{{{\text{OQPSK}}}} } \mathord{\left/ {\vphantom {{TE_{{{\text{OQPSK}}}} } {{\uppi }^{2} }}} \right. \kern-0pt} {{\uppi }^{2} }}$	
		Maximum normalized spectral value	$E_{{{\text{BPSK}}}} {:}E_{{{\text{QPSK}}}}$	/	$E_{{{\text{BPSK}}}} {:}E_{{{\text{OQPSK}}}}$	/	$E_{{{\text{QPSK}}}} {:}E_{{{\text{OQPSK}}}}$	/
			1:1	0.41	1:1	0.81	1:1	1.00
			1:4	0.20	1:4	1.00	1:4	1.00
			4:1	0.41	4:1	0.51	4:1	0.25
14	$\alpha = {2}f_{c} \pm \frac{{1}}{T}$ $f{ = }\frac{1}{4T}$	Theoretical value	${{{2}TE_{{{\text{BPSK}}}} } \mathord{\left/ {\vphantom {{{2}TE_{{{\text{BPSK}}}} } {{{3\uppi }}^{2} }}} \right. \kern-0pt} {{{3\uppi }}^{2} }}$		${{{2}TE_{{{\text{BPSK}}}} } \mathord{\left/ {\vphantom {{{2}TE_{{{\text{BPSK}}}} } {{{3\uppi }}^{2} }}} \right. \kern-0pt} {{{3\uppi }}^{2} }}{ + }{{{2}TE_{{{\text{OQPSK}}}} } \mathord{\left/ {\vphantom {{{2}TE_{{{\text{OQPSK}}}} } {{{3\uppi }}^{2} }}} \right. \kern-0pt} {{{3\uppi }}^{2} }}$		${{{2}TE_{{{\text{OQPSK}}}} } \mathord{\left/ {\vphantom {{{2}TE_{{{\text{OQPSK}}}} } {{{3\uppi }}^{2} }}} \right. \kern-0pt} {{{3\uppi }}^{2} }}$	
		Maximum normalized spectral value	$E_{{{\text{BPSK}}}} {:}E_{{{\text{QPSK}}}}$	/	$E_{{{\text{BPSK}}}} {:}E_{{{\text{OQPSK}}}}$	/	$E_{{{\text{QPSK}}}} {:}E_{{{\text{OQPSK}}}}$	/
			1:1	0.27	1:1	0.54	1:1	0.67
			1:4	0.13	1:4	0.67	1:4	0.67
			4:1	0.27	4:1	0.34	4:1	0.17
15	$$\alpha = {2}f_{c} \pm \frac{{1}}{T}$$$$f{ = }\frac{{1}}{{{2}T}}$$	Theoretical value	0		0		0	
		Maximum normalized spectral value	$E_{{{\text{BPSK}}}} {:}E_{{{\text{QPSK}}}}$	/	$E_{{{\text{BPSK}}}} {:}E_{{{\text{OQPSK}}}}$	/	$E_{{{\text{QPSK}}}} {:}E_{{{\text{OQPSK}}}}$	/
			1:1	0	1:1	0	1:1	0
			1:4	0	1:4	0	1:4	0
			4:1	0	4:1	0	4:1	0
16	$$\alpha = {2}f_{c} \pm \frac{{1}}{T}$$$$f{ = }\frac{{3}}{{{4}T}}$$	Theoretical value	$${{{2}TE_{{{\text{BPSK}}}} } \mathord{\left/ {\vphantom {{{2}TE_{{{\text{BPSK}}}} } {{{5\uppi }}^{2} }}} \right. \kern-0pt} {{{5\uppi }}^{2} }}$$		$${{{2}TE_{{{\text{BPSK}}}} } \mathord{\left/ {\vphantom {{{2}TE_{{{\text{BPSK}}}} } {{{5\uppi }}^{2} }}} \right. \kern-0pt} {{{5\uppi }}^{2} }}{ + }{{{2}TE_{{{\text{OQPSK}}}} } \mathord{\left/ {\vphantom {{{2}TE_{{{\text{OQPSK}}}} } {{{5\uppi }}^{2} }}} \right. \kern-0pt} {{{5\uppi }}^{2} }}$$		$${{{2}TE_{{{\text{OQPSK}}}} } \mathord{\left/ {\vphantom {{{2}TE_{{{\text{OQPSK}}}} } {{{5\uppi }}^{2} }}} \right. \kern-0pt} {{{5\uppi }}^{2} }}$$	
		Maximum normalized spectral value	$E_{{{\text{BPSK}}}} {:}E_{{{\text{QPSK}}}}$	/	$E_{{{\text{BPSK}}}} {:}E_{{{\text{OQPSK}}}}$	/	$E_{{{\text{QPSK}}}} {:}E_{{{\text{OQPSK}}}}$	/
			1:1	0.16	1:1	0.32	1:1	0.40
			1:4	0.08	1:4	0.40	1:4	0.40
			4:1	0.16	4:1	0.20	4:1	0.10
17	$\alpha = {2}f_{c} \pm \frac{{1}}{T}$ $f{ = }\frac{{1}}{T}$	Theoretical value	${{TE_{{{\text{BPSK}}}} } \mathord{\left/ {\vphantom {{TE_{{{\text{BPSK}}}} } {{{3\uppi }}^{2} }}} \right. \kern-0pt} {{{3\uppi }}^{2} }}$		${{TE_{{{\text{BPSK}}}} } \mathord{\left/ {\vphantom {{TE_{{{\text{BPSK}}}} } {{{3\uppi }}^{2} }}} \right. \kern-0pt} {{{3\uppi }}^{2} }}{ + }{{TE_{{{\text{OQPSK}}}} } \mathord{\left/ {\vphantom {{TE_{{{\text{OQPSK}}}} } {{{3\uppi }}^{2} }}} \right. \kern-0pt} {{{3\uppi }}^{2} }}$		${{TE_{{{\text{OQPSK}}}} } \mathord{\left/ {\vphantom {{TE_{{{\text{OQPSK}}}} } {{{3\uppi }}^{2} }}} \right. \kern-0pt} {{{3\uppi }}^{2} }}$	
		Maximum normalized spectral value	$E_{{{\text{BPSK}}}} {:}E_{{{\text{QPSK}}}}$	/	$E_{{{\text{BPSK}}}} {:}E_{{{\text{OQPSK}}}}$	/	$E_{{{\text{QPSK}}}} {:}E_{{{\text{OQPSK}}}}$	/
			1:1	0.14	1:1	0.27	1:1	0.33
			1:4	0.07	1:4	0.33	1:4	0.33
			4:1	0.14	4:1	0.17	4:1	0.08
18	$\alpha = {2}f_{c} \pm \frac{{2}}{T}$ $f{ = 0}$	Theoretical value	0		0		/	
		Maximum normalized spectral value	$E_{{{\text{BPSK}}}} {:}E_{{{\text{QPSK}}}}$	/	$E_{{{\text{BPSK}}}} {:}E_{{{\text{OQPSK}}}}$	/	$E_{{{\text{QPSK}}}} {:}E_{{{\text{OQPSK}}}}$	/
			1:1	0	1:1	0	1:1	/
			1:4	0	1:4	0	1:4	/
			4:1	0	4:1	0	4:1	/
19	$$\alpha = {2}f_{c} \pm \frac{{2}}{T}$$$$f{ = }\frac{{1}}{{{4}T}}$$	Theoretical value	$${{{2}TE_{{{\text{BPSK}}}} } \mathord{\left/ {\vphantom {{{2}TE_{{{\text{BPSK}}}} } {{{15\uppi }}^{2} }}} \right. \kern-0pt} {{{15\uppi }}^{2} }}$$		$${{{2}TE_{{{\text{BPSK}}}} } \mathord{\left/ {\vphantom {{{2}TE_{{{\text{BPSK}}}} } {{{15\uppi }}^{2} }}} \right. \kern-0pt} {{{15\uppi }}^{2} }}$$		/	
		Maximum normalized spectral value	$E_{{{\text{BPSK}}}} {:}E_{{{\text{QPSK}}}}$	/	$E_{{{\text{BPSK}}}} {:}E_{{{\text{OQPSK}}}}$	/	$E_{{{\text{QPSK}}}} {:}E_{{{\text{OQPSK}}}}$	/
			1:1	0.05	1:1	0.05	1:1	/
			1:4	0.03	1:4	0.03	1:4	/
			4:1	0.05	4:1	0.05	4:1	/
20	$$\alpha = {2}f_{c} \pm \frac{{2}}{T}$$$$f{ = }\frac{{1}}{{{2}T}}$$	Theoretical value	$${{{2}TE_{{{\text{BPSK}}}} } \mathord{\left/ {\vphantom {{{2}TE_{{{\text{BPSK}}}} } {{{3\uppi }}^{2} }}} \right. \kern-0pt} {{{3\uppi }}^{2} }}$$		$${{{2}TE_{{{\text{BPSK}}}} } \mathord{\left/ {\vphantom {{{2}TE_{{{\text{BPSK}}}} } {{{3\uppi }}^{2} }}} \right. \kern-0pt} {{{3\uppi }}^{2} }}$$		/	
		Maximum normalized spectral value	$E_{{{\text{BPSK}}}} {:}E_{{{\text{QPSK}}}}$	/	$E_{{{\text{BPSK}}}} {:}E_{{{\text{OQPSK}}}}$	/	$E_{{{\text{QPSK}}}} {:}E_{{{\text{OQPSK}}}}$	/
			1:1	0.27	1:1	0.27	1:1	/
			1:4	0.13	1:4	0.13	1:4	/
			4:1	0.27	4:1	0.27	4:1	/
21	$$\alpha = {2}f_{c} \pm \frac{{2}}{T}$$$$f{ = }\frac{{3}}{{{4}T}}$$	Theoretical value	$${{{2}TE_{{{\text{BPSK}}}} } \mathord{\left/ {\vphantom {{{2}TE_{{{\text{BPSK}}}} } {{{7\uppi }}^{2} }}} \right. \kern-0pt} {{{7\uppi }}^{2} }}$$		$${{{2}TE_{{{\text{BPSK}}}} } \mathord{\left/ {\vphantom {{{2}TE_{{{\text{BPSK}}}} } {{{7\uppi }}^{2} }}} \right. \kern-0pt} {{{7\uppi }}^{2} }}$$		/	
		Maximum normalized spectral value	$E_{{{\text{BPSK}}}} {:}E_{{{\text{QPSK}}}}$	/	$E_{{{\text{BPSK}}}} {:}E_{{{\text{OQPSK}}}}$	/	$E_{{{\text{QPSK}}}} {:}E_{{{\text{OQPSK}}}}$	/
			1:1	0.12	1:1	0.12	1:1	/
			1:4	0.06	1:4	0.06	1:4	/
			4:1	0.12	4:1	0.12	4:1	/
22	$\alpha = {2}f_{c} \pm \frac{{2}}{T}$ $f{ = }\frac{{1}}{T}$	Theoretical value	0		0		/	
		Maximum normalized spectral value	$E_{{{\text{BPSK}}}} {:}E_{{{\text{QPSK}}}}$	/	$E_{{{\text{BPSK}}}} {:}E_{{{\text{OQPSK}}}}$	/	$E_{{{\text{QPSK}}}} {:}E_{{{\text{OQPSK}}}}$	/
			1:1	0	1:1	0	1:1	/
			1:4	0	1:4	0	1:4	/
			4:1	0	4:1	0	4:1	/
23	$$\alpha { < 2}f_{c} { - }\frac{{2}}{T}$$$$\alpha { > 2}f_{c} { + }\frac{{2}}{T}$$	Theoretical value	$$\ll {{TE} \mathord{\left/ {\vphantom {{TE} {{\uppi }^{2} }}} \right. \kern-0pt} {{\uppi }^{2} }}$$		$$\ll {{TE} \mathord{\left/ {\vphantom {{TE} {{\uppi }^{2} }}} \right. \kern-0pt} {{\uppi }^{2} }}$$		$$\ll {{TE} \mathord{\left/ {\vphantom {{TE} {{\uppi }^{2} }}} \right. \kern-0pt} {{\uppi }^{2} }}$$	
		Maximum normalized spectral value	$E_{{{\text{BPSK}}}} {:}E_{{{\text{QPSK}}}}$	/	$E_{{{\text{BPSK}}}} {:}E_{{{\text{OQPSK}}}}$	/	$E_{{{\text{QPSK}}}} {:}E_{{{\text{OQPSK}}}}$	/
			1:1	$\ll {0}{\text{.1}}$	1:1	$\ll {0}{\text{.1}}$	1:1	$\ll {0}{\text{.1}}$
			1:4	$\ll {0}{\text{.1}}$	1:4	$\ll {0}{\text{.1}}$	1:4	$\ll {0}{\text{.1}}$
			4:1	$\ll {0}{\text{.1}}$	4:1	$\ll {0}{\text{.1}}$	4:1	$\ll {0}{\text{.1}}$

**Figure 6:**
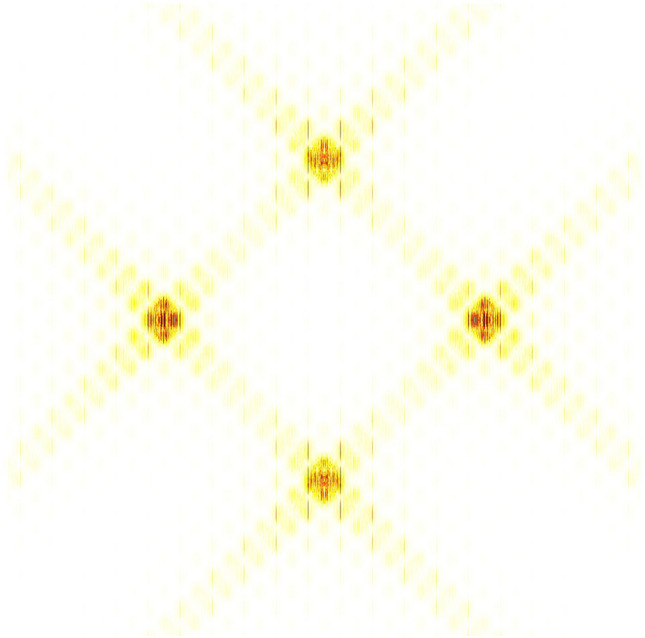
Grayscale projection of BPSK + QPSK cyclic spectrum.

As can be seen from Table 1, the cyclic spectrum contains abundant individual characteristics of mixed signals, so it is an effective recognition feature. The problem of one-dimensional signal modulation recognition is transformed into that of two-dimensional image recognition. Here, two-dimensional grayscale projection is used to represent the characteristics of the cyclic spectrum, and the corresponding two-dimensional grayscale projection of Fig. 5 is shown in Fig. 6.

**Figure 7:**
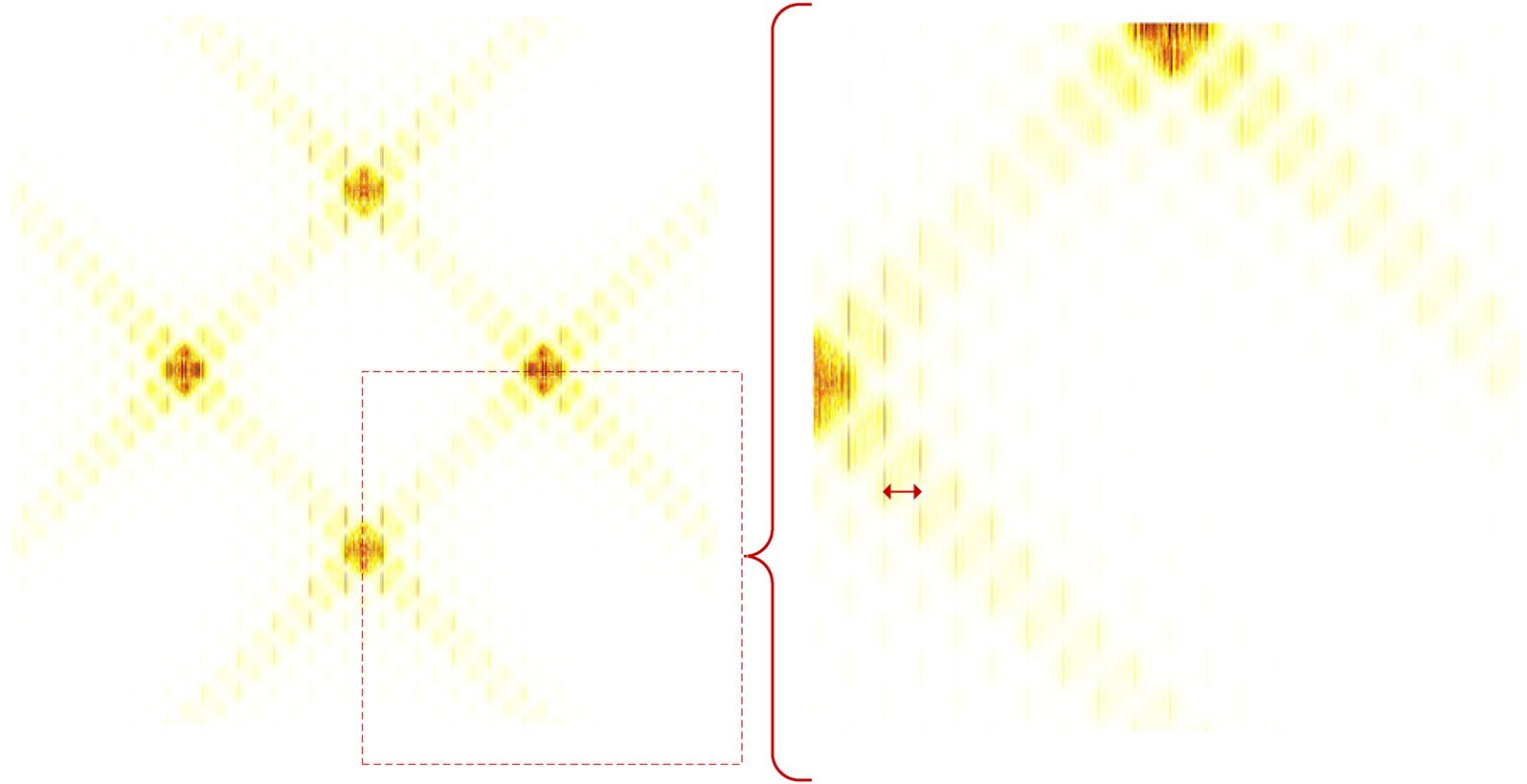
Part area ($\alpha { < }0{, }f \le 0$) of grayscale projection image.

It can be seen from the cyclic spectrum equation and Fig. 6 that the cyclic spectrum features are symmetrical. In order to reduce the amount of calculation, part of its region ($\alpha { < }0{, }f \le 0$) is taken as the recognition feature domain, as shown in Fig. 7.

## Image feature enhancement

From the cyclic spectrum of mixed signals and Table 1, it can be seen that the effective recognition features exist at a certain type of frequency, while the features in Fig. 6 and Fig. 7 are chaotic, with more redundant information, and the characteristic values of mixed signals with different energy ratios are different. In order to enhance the readability and consistency of image features, a new nonlinear piecewise mapping method is proposed to preprocess images. In addition, according to the mixed signal cyclic spectrum equation, when the receiver intermediate frequency(IF) ($f_{c}$) is fixed, the symbol rate (${{1} \mathord{\left/ {\vphantom {{1} T}} \right. \kern-0pt} T}$) is the key factor affecting the distribution of the recognition features in the projection map, as shown by the red double arrows in Fig. 7. In order to enhance the adaptability of the recognition feature field to the change of the symbol rate, a directed pseudo-clustering method is proposed to eliminate the impact of the change of the symbol rate.

### Nonlinear piecewise mapping

**Figure 8:**
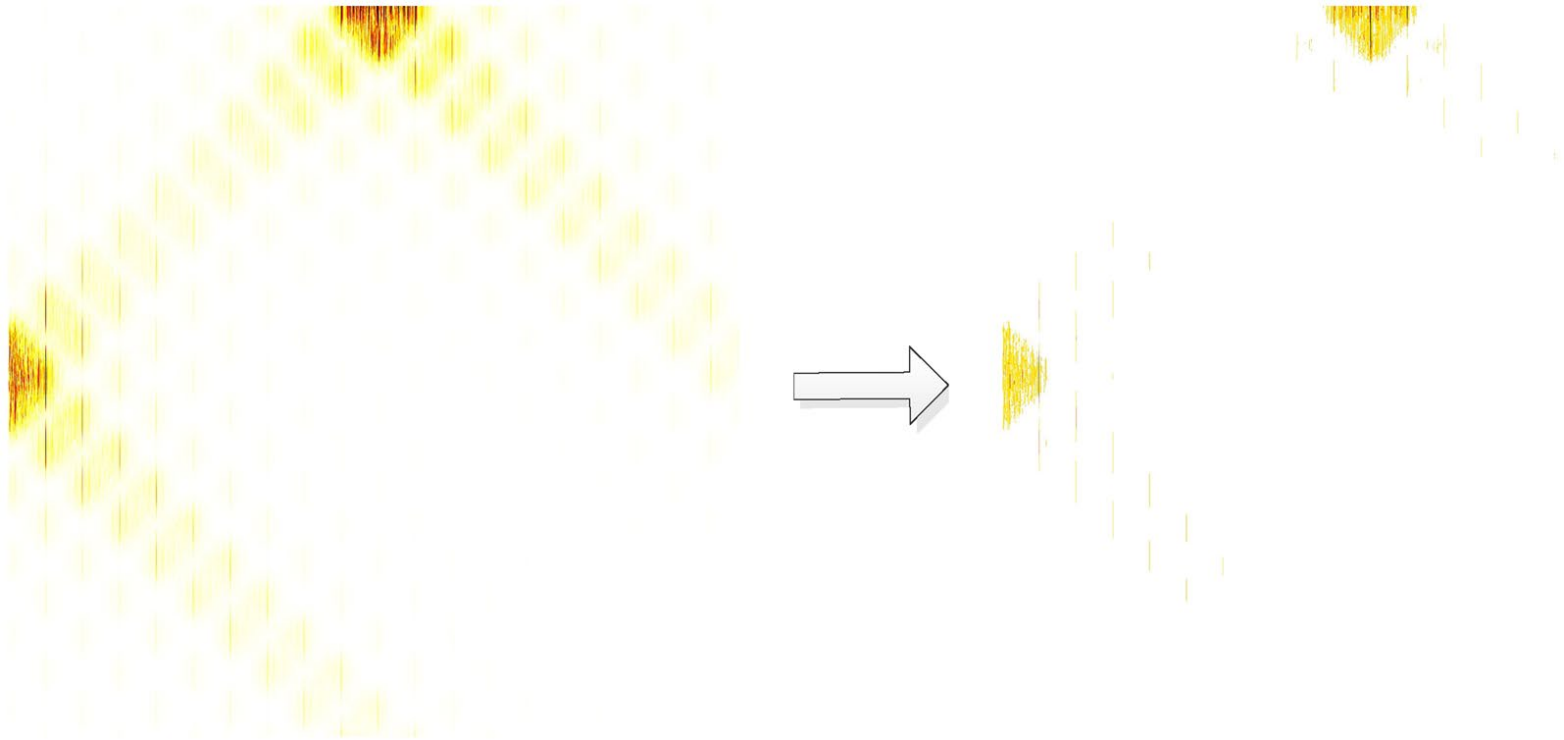
Piecewise map of BPSK + QPSK signal cyclic spectrum projection.

The purpose of nonlinear piecewise mapping is to approximate the consistency of signal characteristics with different energy ratios, reduce the computational cost of subsequent recognition networks, and improve the efficiency of recognition networks. For the grayscale image with maximum normalization, the nonlinear piecewise mapping method follows the following principles:
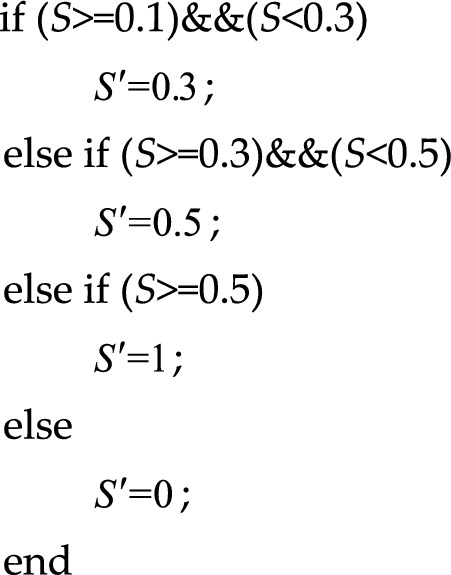


In the above mapping, $S$ represents the original normalized cyclic spectrum value, and $S^{\prime}$ represents the mapped spectrum value. The above processing process is shown in Fig. 8, and the figure on the right is the result after processing. It can be seen from the figure that the difference characteristics of signal have been significantly enhanced.

### Directed pseudo-clustering

**Figure 9:**
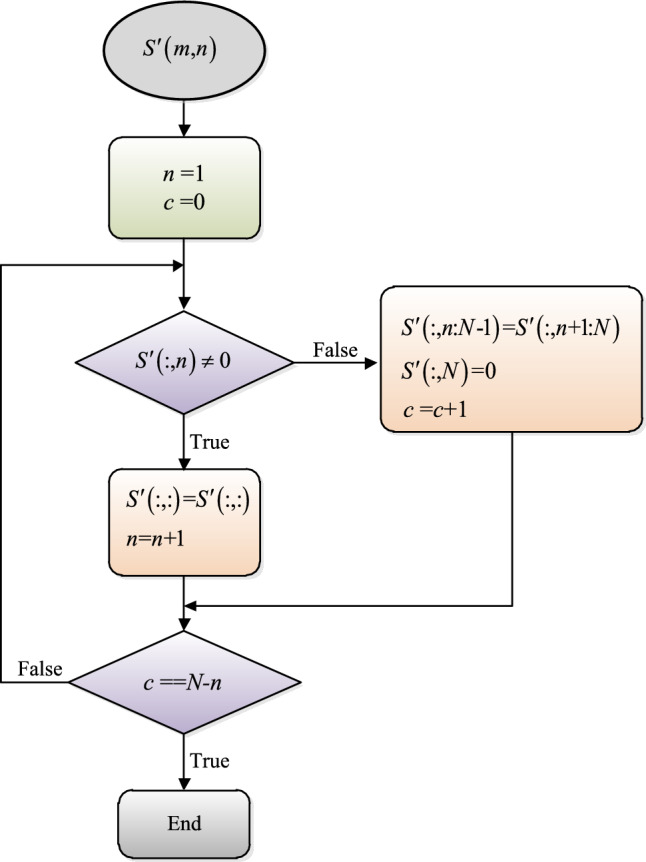
Processing flow of directed pseudo-clustering method.

The purpose of directed pseudo-clustering is to eliminate the spectral line spacing caused by the symbol rate. Because it draws lessons from the idea of clustering, it also has the requirement of direction, so it is called directed pseudo-clustering. It is assumed that the cyclic spectrum projection after nonlinear piecewise mapping is represented by $S^{\prime}\left( {m{,}n} \right)$, where $m\;{ = }\;1{,}2{,} \ldots M{,}\;\; \, n\;{ = }\;1{,}2{,} \ldots N$, whose directed pseudo-clustering processing flow is shown in Fig. 9.

**Figure 10:**
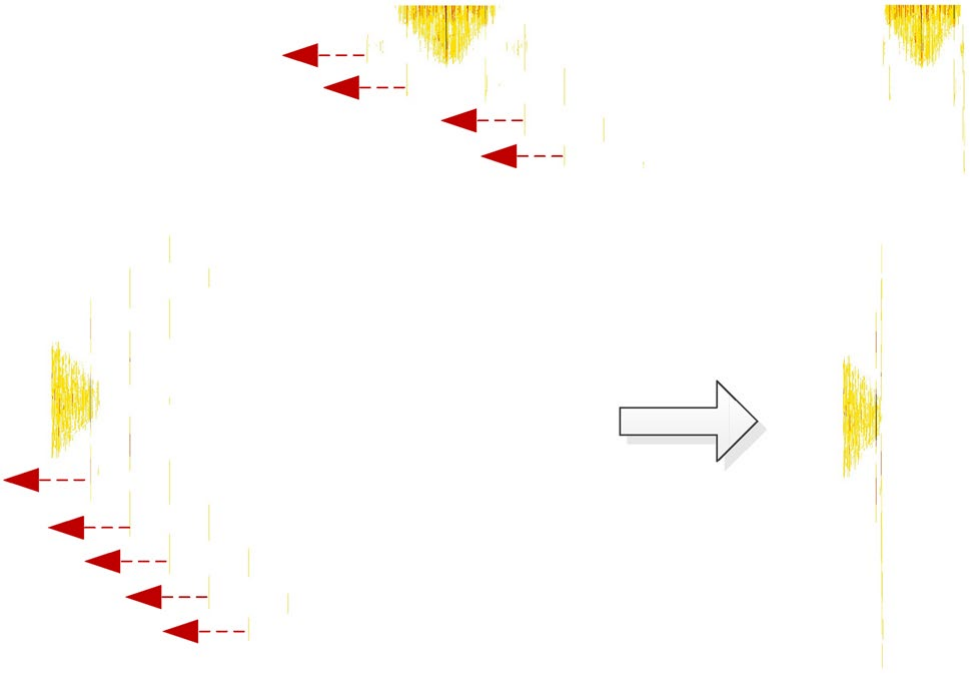
Schematic diagram of directed pseudo-clustering processing.

The processing process of Fig. 9 is shown in Fig. 10, and the right figure shows the results after processing. As can be seen from the figure, all the spectral lines in Fig. 8 converge towards the direction of the zero cyclic frequency axis, eliminating the spectral line spacing caused by the symbol rate, so the characteristic image is not affected by the change of symbol rate.

**Figure 11:**
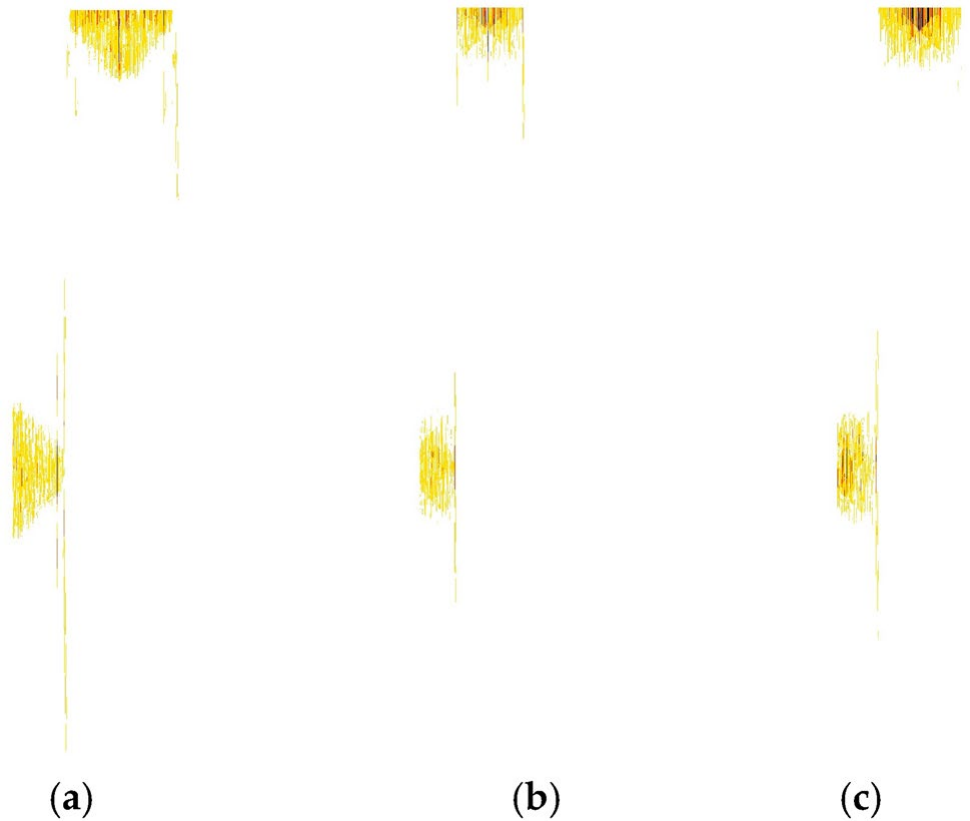
Cyclic spectrum projection feature enhancement image of three kinds of mixed double-signals. (**a**) BPSK + QPSK. (**b**) BPSK + OQPSK. (**c**) QPSK + OQPSK.

Figure 11 shows the enhanced grayscale projection image of cyclic spectrum when the energy of three types of mixed signals is equal. It can be seen from the figure that there are obvious differences between the three types of signals. The grayscale image is an effective recognition feature, which can be used for modulation recognition of mixed double-signals.

## Deep residual neural network

ResNet is a kind of network structure proposed in 2015. It refers to adding the idea of residual learning into the traditional convolutional neural network, which solves the problems of gradient dispersion and accuracy decline (training set) in the deep network.

**Figure 12:**
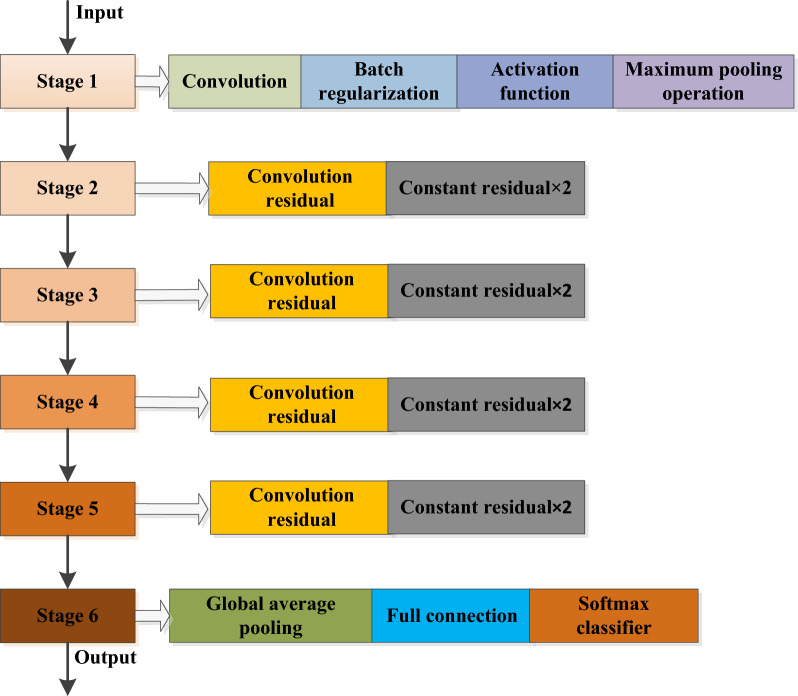
Structure diagram of ResNet50.

After years of development, ResNet has derived a variety of network structures with different convolution layer depths, such as ResNet50 and ResNet101. This paper adopts ResNet50 structure, which is mainly composed of multiple residual modules^25,30^, and its structure is shown in Fig. 12. The ResNet50 network structure consists of one full connection layer and 49 convolution layers, and the network model runs in six stages. The first stage includes convolution, batch regularization, activation function and maximum pooling operation. The second to fifth stages include convolution residual module and constant residual module. The sixth stage includes the global average pooling layer operation and the softmax classifier for the full connection layer.

In this paper, the modulation recognition based on ResNet is a supervised learning method. The input of the network in Fig. 12 is the grayscale image after the enhanced cyclic spectrum projection of the three types of mixed signals as shown in Fig. 11. The grayscale image has undergone multi-stage convolution and pooling operations of the residual network. Finally, the network carries out nonlinear modulation type mapping according to its characteristics, and outputs the corresponding three modulation types of mixed signals.

## Simulation test and analysis

In order to verify the effectiveness of the proposed method, a simulation evaluation is carried out based on MATLAB software platform and HP Z840 workstation. In this paper, we simulate and generate three types of mixed signals, i.e., BPSK + QPSK, BPSK + OQPSK and OQPSK, to verify the performance of the proposed method. The parameters of simulation signals are set as follows: intermediate frequency is 150 kHz, symbol rate is 50 k bit/s, sampling frequency is 1.2 MHz^34,35^. The signals are all formed by sine–cosine rolling drop method, the forming coefficient is 0.35, and the number of signal points is 8192.

In the experiment, we resize the input images to a fixed size of 224*224*3, which matched the input shape of the original ResNet50 network model. So the network parameters of ResNet50 in the experiment are consistent with the original model. In order to train the network, we simulate and generate 1000 signals for each type of mixed signal, and use their enhanced cyclic spectrum graphs as training samples. There are a total of 3000 training samples for the three types of mixed signals for training. All the training samples are noise-free, and the energy ratio of the mixed signals is fixed at 1:1. We use these training data to train the network, and test the trained network with different test data to evaluate the performance of the proposed method.

### Analysis of the influence of noise on the performance of the method

In order to have a clearer and more specific understanding of the recognition, the simulation analysis of the impact of noise on each type of mixed signal was carried out.

**Figure 13:**
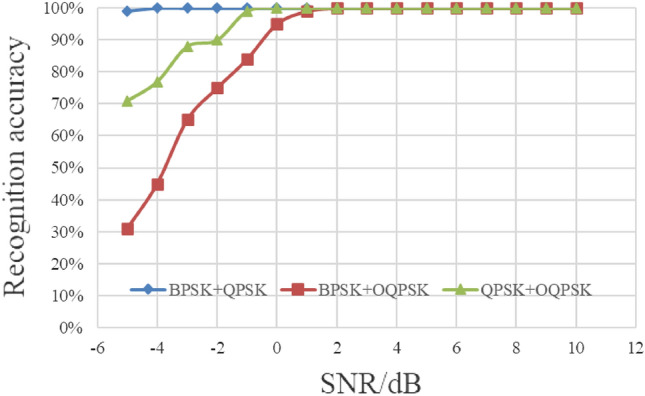
Recognition results under different SNR.

In this section, we set the energy ratio of each type of mixed signal to 1:1, and the signal-to-noise ratio (SNR) ranges from − 5 to 10 dB with a step of 1 dB. For each SNR, there are 200 cyclic spectrum graphs on each signal type, and a total of 9600 graphs for the three types of mixed signals are used as test samples. The test results are shown in Fig. 13.

As can be seen from the Fig. 13, when SNR is no less than 0 dB, the recognition rate is greater than 95%. Among the above three kinds of mixed signals, BPSK + QPSK signal has significantly better recognition performance than other signals. The reason is that the individual characteristics of the mixed signals with the same energy are the most obvious after enhanced processing. In addition, experimental results show that the proposed method has good generalization and noise suppression ability.

### Analysis of the influence of energy ratio on the performance of the method

**Figure 14:**
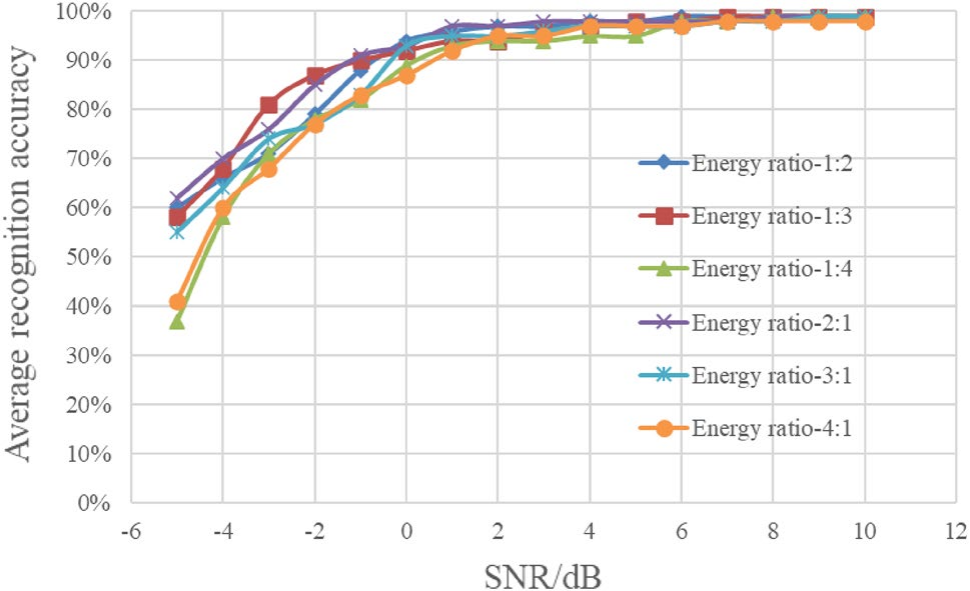
Recognition results of different energy ratios.

For mixed signals, different energy ratios can lead to different characteristics of their mixed features, so the energy ratio is the most important factor affecting the recognition of mixed signals. This section investigates the influence of different energy ratios on the method. On the basis of the test samples in section “Analysis of the influence of noise on the performance of the method”, the test samples under the different energy ratios of 1:2, 2:1, 1:3, 3:1, 1:4 and 4:1 are added. A total of 57,600 samples are collected. The average recognition results of three types of mixed signals under different energy ratios are shown in Fig. 14.

As can be seen from Fig. 14, the average recognition rate of three types of mixed signals is slightly different when the energy ratio is different. On the whole, when the SNR is greater than − 2 dB, the average recognition rate of the six energy ratio cases is greater than 90%. It shows that nonlinear piecewise mapping in feature enhancement weakens the influence of energy ratio to a certain extent, and effectively improves the adaptability of the algorithm.

**Figure 15:**
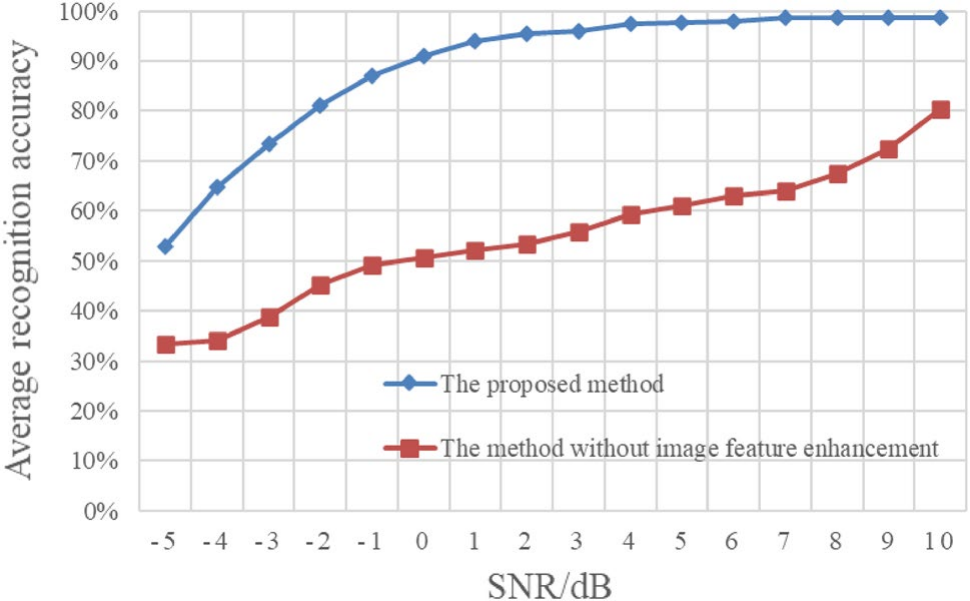
Average recognition results of different energy ratios without image feature enhancement.

To further demonstrate the effectiveness of the proposed image feature enhancement method under different energy ratios, we present the experimental results without image enhancement in Fig. 15. In this experiment, both the training samples and the test samples of the compared method are the original cyclic spectrum graphs without image enhancement. The rest of the experimental settings are consistent with those in Fig. 14. The experimental results in Fig. 15 are the average recognition results of the three types of mixed signals under different energy ratios. It can be seen from the Fig. 15 that the recognition rate of the mixed signals is significantly improved after image enhancement, which proves that the proposed image feature enhancement algorithm can effectively enhance the feature separability of cyclic spectrum graph.

### Analysis of the influence of symbol rate on the performance of the method

**Figure 16:**
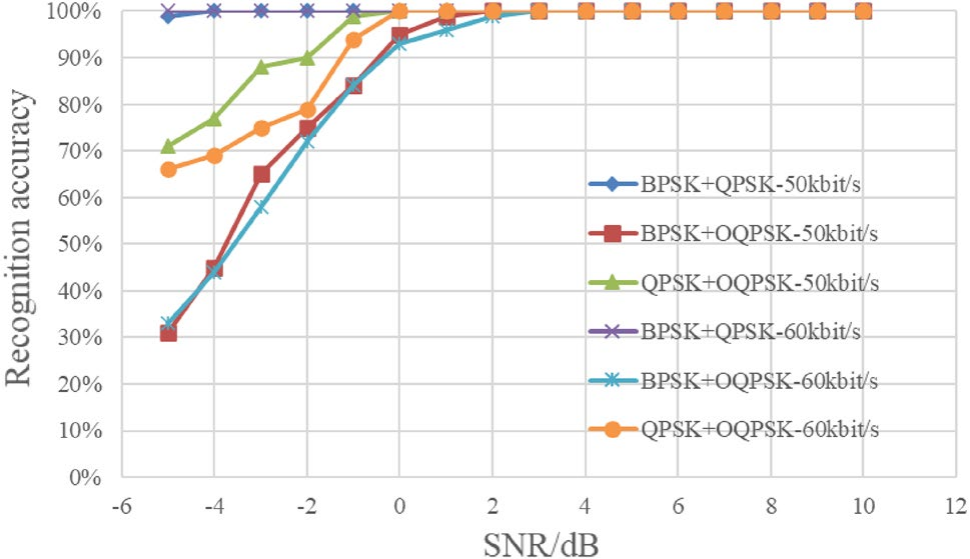
Recognition results of different symbol rates.

In the process of signal reception, RF signal is down converted to fixed IF by the receiver, and then the symbol rate of the signal becomes one of the main factors affecting the performance of modulation recognition. This section analyzes the influence of different symbol rates on the method. In this section, the symbol rate of the test samples is changed to 60 k bit/s, and the other parameter settings of the test samples are consistent with those in section “Analysis of the influence of noise on the performance of the method”. The results are shown in Fig. 16.

It can be seen from Fig. 16 that the recognition performance is roughly equivalent under different symbol rates, which indicates that the method in this paper is insensitive to symbol rate transformation, and also indirectly proves the effectiveness of the directed pseudo-clustering method in feature enhancement. The proposed method has good robustness and adaptability.

**Figure 17:**
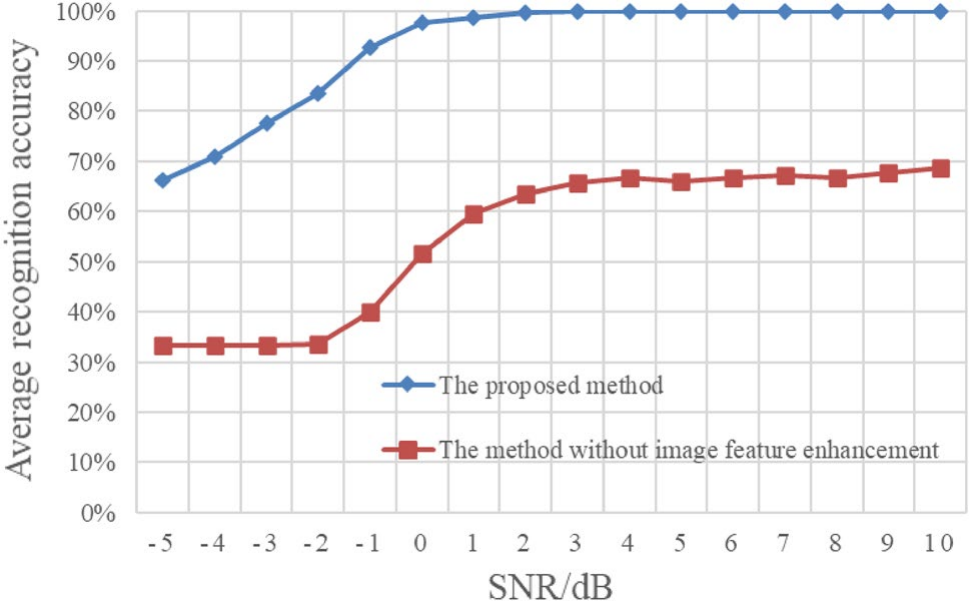
Average recognition results of different symbol rates without image feature enhancement.

To further demonstrate the effectiveness of the proposed image feature enhancement method under different symbol rates, we also present the experimental results without image enhancement in Fig. 17. In this experiment, both the training samples and the test samples are the original cyclic spectrum graphs without image enhancement. The rest of the experimental settings are consistent with those in Fig. 16. The experimental results in Fig. 17 are the average recognition results of the three types of mixed signals under different symbol rates. As shown in Fig. 17, image enhancement method leads to a remarkable improvement in the recognition rate of the mixed signals.

### Performance comparison with existing methods

**Figure 18:**
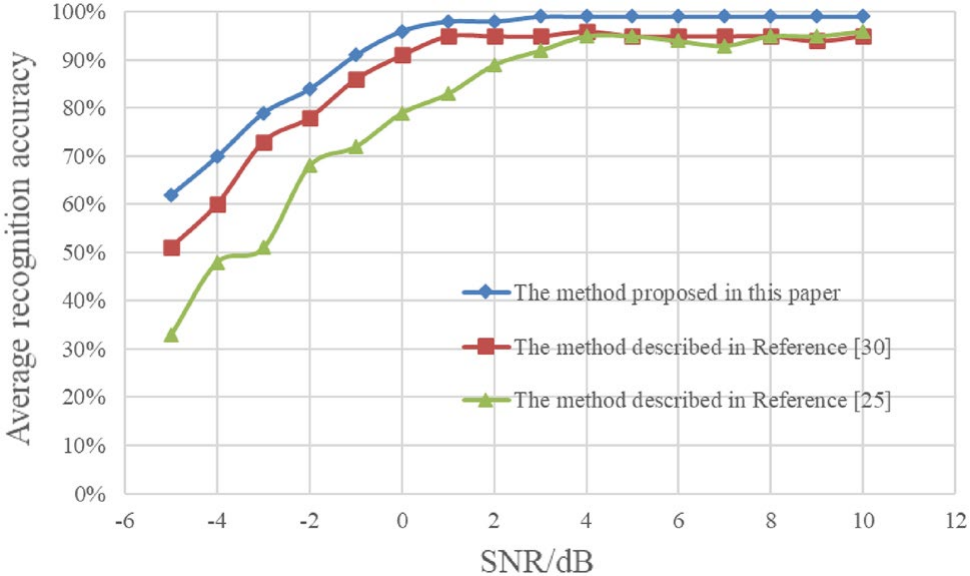
Average recognition results of different methods.

In order to have a more comprehensive evaluation of the method, this section compares the method in this paper with the reference^25^ and^30^. The training samples in this paper are used to train the methods proposed in^25^ and^30^. The test samples include all the samples in sections “Analysis of the influence of noise on the performance of the method”–“Analysis of the influence of symbol rate on the performance of the method”. Figure 18 shows the average recognition results of the three methods with different energy ratios and symbol rates.

According to Fig. 18, the recognition performance of reference^30^ is better than that of reference^25^ at low SNR, because the cyclic spectrum after dimension reduction has good anti-noise characteristics. In the whole SNR range, the recognition results of the proposed method are superior to those in^25^ and^30^. ResNet is used in all three methods, indicating that the feature domain and feature enhancement method in this paper play a key role, and the proposed method has better recognition performance and adaptability.

## Conclusion

Based on the insensitivity of cyclic spectrum projection to noise, a mixed signal recognition feature domain is constructed, and two new methods, namely nonlinear piecewise mapping and directed pseudo-clustering, are proposed to enhance the feature domain from different dimensions. On this basis, ResNet50 is used to carry out in-depth intelligent feature analysis and classification of the feature domain. Three kinds of common mixed double-signals in satellite communication are recognized effectively, and the simulation results prove the effectiveness of this method. In addition, ResNet50 model is mature and widely used, so the method in this paper is easy to implement in engineering. In this paper, the interference of adjacent channels is mainly considered, and the main form is the mixture of two modulation types of signals. For three or more mixed signals, further research will be conducted in the follow-up work.

## Data Availability

The datasets used during the current study available from the corresponding author on reasonable request.
